# Structure-activity relationship of pyrazole-based corrosion inhibitors for carbon steel in HCl: combined experimental and theoretical study

**DOI:** 10.1038/s41598-026-61895-8

**Published:** 2026-07-19

**Authors:** Basma M. A. Khedr, Sayed K. Ramadan, Sherine A. Abdelkader, Samar Abdelhamed, Mona A. El-Etre, Magdy A. M. Ibrahim, Reham H. Tammam, Ahmed Nasser

**Affiliations:** 1 Chemistry Department, Faculty of Science, Ain Shams University, Cairo, 11566 Egypt; 2 Basic Engineering Sciences Department, Faculty of Engineering, Benha University, Benha, Egypt; 3 Department of Basic Science, Faculty of Engineering at Shoubra, Benha University, Benha, Egypt; 4 Department of Chemistry, Faculty of Science, Cairo University, Giza, 12613 Egypt; 5 Faculty of Industry and Energy Technology, New Cairo Technological University, New Cairo, 11835 Egypt; 6 The Higher Institute of Engineering, New Elmarg, El-Qalyubia, Egypt

**Keywords:** Carbon steel, Pyrazole derivatives, EIS, DFT, Monte Carlo simulation, Adsorption isotherm, Chemistry, Materials science

## Abstract

The corrosion inhibition performance of two newly synthesized pyrazole-based derivatives, **P6** (bis-pyrazole) and **P8** (pyrazolyl-thiadiazine), toward carbon steel in 1.0 M HCl was systematically investigated using combined experimental and theoretical approaches. Electrochemical and gravimetric results revealed high inhibition efficiencies exceeding 95% at 5 × 10⁻⁴ M, with **P6** exhibiting superior performance. This enhanced performance is attributed to its stronger adsorption affinity and more stable interaction with the steel surface. Polarization measurements revealed predominantly anodic inhibition behavior, although both anodic and cathodic reactions were affected, while impedance analysis confirmed the formation of a protective adsorbed film. Surface characterization (SEM, EDX, and AFM) further supported the development of a compact and homogeneous protective layer. Thermodynamic analysis suggested a mixed adsorption mechanism involving electrostatic and chemical interactions. Computational studies (DFT and Monte Carlo simulations) corroborated the experimental findings, demonstrating strong adsorption on the Fe (110) surface.

**Supplementary Information:**

The online version contains supplementary material available at 10.1038/s41598-026-61895-8.

## Introduction

Corrosion of metallic materials is a major challenge across industrial sectors, causing significant economic losses, safety risks, and operational inefficiencies^1,2^. Structures and facilities in sectors such as petrochemicals, chemical processing, wastewater treatment, and marine operations are particularly exposed due to persistent contact with aggressive environments^3,4^. Among structural materials, carbon steel (CS) is widely used owing to its outstanding mechanical properties, abundance, and cost-effectiveness. However, its susceptibility to corrosion, especially in acidic and chloride-containing media, presents a significant challenge for long-term durability. In industrial applications such as descaling, acid pickling, oil well acidizing, and chemical cleaning, carbon steel frequently encounters hydrochloric acid, which rapidly disrupts its protective oxide layer and accelerates metal dissolution^5,6^. While complete elimination of corrosion is unfeasible, its progression can be effectively controlled using various strategies, including material modification, cathodic protection, surface treatments, and the application of corrosion inhibitors.

Among available strategies, organic inhibitors stand out as a cost-effective and sustainable means of limiting acid corrosion. Organic inhibitors act by adsorbing on the metal surface and forming a protective film that reduces both anodic metal dissolution and cathodic hydrogen evolution. Their efficiency strongly depends on molecular structure, electron-rich functional groups, and the ability to interact with the metal surface. Compounds containing heteroatoms such as nitrogen and sulfur, in combination with conjugated π-electron systems, often show superior adsorption capabilities due to enhanced electron-donating properties and strong affinity for the vacant d-orbitals of iron^7–9^. Pyrazole and pyrazolone derivatives have recently attracted attention as a promising class of metal corrosion inhibitors, largely owing to the existence of π-electrons in the -C = N- azomethine moiety, which can interact with metal surfaces and form stable complexes. Heterocyclic compounds with pyrazole moieties have emerged as promising inhibitors for CS in different media^10–13^.

These molecules feature versatile architectures, multiple nitrogen donor sites, and conjugated π-systems, which promote strong surface adsorption and stable film formation. The inhibition performance is influenced by electronic distribution, molecular planarity, and spatial arrangement, which together govern the interaction strength between the inhibitor and the metal^14^. Additionally, the inclusion of other heterocycles or sulfur-containing groups can further enhance surface coverage through synergistic effects. Recent research highlights the importance of combining experimental techniques with theoretical modeling to understand molecular-level inhibition mechanisms. Rashid et al.^15^ reported that a pyrazole derivative acts as an efficient mixed-type inhibitor for carbon steel in 1.0 M HCl based on combined experimental and theoretical analyses. Also, Sayed et al.^16^ demonstrated the effectiveness of pyrazole and pyrazolone derivatives for corrosion inhibition, highlighting the influence of molecular structure on performance.

In recent years, several classes of organic compounds, including Schiff bases, triazoles, imidazoles, quinoxalines, and pyridine derivatives, have been widely studied as corrosion inhibitors for carbon steel in acidic media, with performance strongly influenced by molecular structure and adsorption behavior^17–22^. Among these systems, pyrazole-based compounds have attracted particular attention due to their rich electron density and multiple adsorption centers. However, the effect of molecular architecture, especially the incorporation of additional adsorption sites and sulfur-containing heterocycles, remains insufficiently understood, highlighting the need for clearer structure–activity relationships for rational inhibitor design in industrial environments such as oil and gas production, acid pickling, chemical cleaning, and wastewater treatment, where corrosion directly impacts cost and operational safety.

Despite advances in pyrazole-based inhibitors, comparative studies under identical experimental conditions remain limited. In particular, the influence of multiple pyrazole units versus sulfur-containing frameworks on adsorption behavior, inhibition efficiency, and thermal stability is not yet fully clarified. To address this gap, this work investigates two newly synthesized inhibitors, a bis-pyrazole (**P6**) and a pyrazolyl-thiadiazine (**P8**), for carbon steel in 1.0 M HCl. Their performance was evaluated using weight-loss, potentiodynamic polarization, and EIS, supported by DFT calculations and Monte Carlo simulations. A structured comparison table was also used to ensure consistent evaluation across molecular systems. This study provides a direct structure–activity correlation and mechanistic insight into adsorption and protective film formation, supporting the rational design of efficient corrosion inhibitors for aggressive acidic media.

## Experimental details

### Synthesis and characterization of inhibitors

**Scheme 1:**
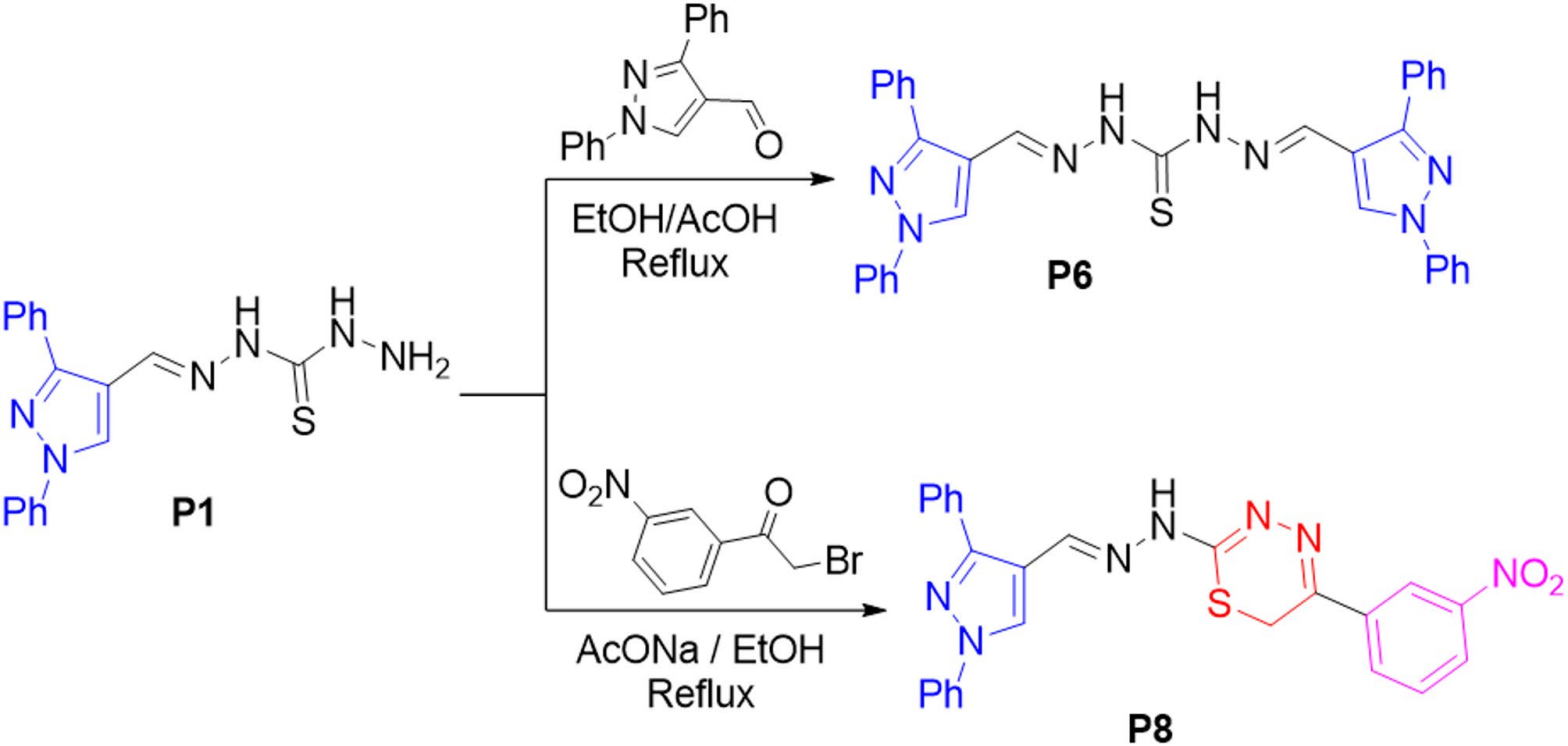
Synthesis of bis-pyrazole (**P6**) and pyrazolyl-thiadiazine derivative (**P8**).

The investigated pyrazole-based inhibitors, namely the bis-pyrazole (**P6**) and the pyrazolyl-thiadiazine (**P8**), were synthesized from pyrazolyl-thiocarbohydrazone **P1**^23^ using standard procedures. Detailed experimental conditions, synthetic routes, and full spectroscopic characterization (FT-IR, ¹H NMR, ¹³C NMR, and elemental analysis) are provided in the Supplementary Information (Section SI (1)). The synthetic pathways of the investigated compounds are illustrated in Scheme 1, while the corresponding spectroscopic data are presented in Figs. S1 and S2.

### Materials and test solutions

Carbon steel (CS) specimens used in this study contained (wt%) 94.00% Fe, 0.08% C, 1.00% Mn, 0.60% Si, 2.00% Cr, 1.20% Ni, 0.03% P, 0.02% S, 0.73% O, and 0.34% N. Carbon steel was selected because of its extensive industrial applications and its susceptibility to corrosion in acidic environments. Prior to use, the steel coupons were mechanically polished using emery papers of progressively finer grades (400–2500 grit) to obtain a smooth, mirror-like surface. The specimens were then rinsed thoroughly with deionized water, degreased with acetone, and dried before immersion in the test solutions. Stock solutions of the synthesized pyrazole derivatives (**P6** and **P8**) were prepared in 1.0 M HCl, a commonly used aggressive medium for evaluating corrosion inhibitors and simulating industrial acid-cleaning conditions. Stock solutions were prepared at a concentration of 1 × 10⁻² M, and working solutions were obtained by serial dilution to achieve final concentrations in the range of 5 × 10⁻⁶ to 5 × 10⁻⁴ M. This concentration range was selected to investigate the concentration-dependent inhibition performance of the synthesized compounds. All experiments were initially conducted at ambient temperature (25 ± 1 °C), while additional measurements at elevated temperatures (25–50 °C) were performed to evaluate the effect of temperature on inhibitor performance and adsorption behavior.

### Gravimetric and electrochemical measurements

#### Weight loss measurements

Gravimetric measurements were performed to evaluate the corrosion inhibition performance of pyrazole candidates **P6** and **P8** on CS in 1.0 M HCl. CS coupons (1.0 × 1.0 × 0.7 cm) were mechanically polished, rinsed with deionized water and acetone, and then immersed in acidic solutions containing different concentrations of the inhibitors. The specimens were exposed for various immersion times up to 6 h at room temperature. After each interval, the coupons were removed, cleaned, dried, and reweighed.

#### Electrochemical measurement (EMs)

Electrochemical measurements were performed using a potentiostat/galvanostat system (Origalys) equipped with a conventional three-electrode cell. CS was used as the working electrode, while a platinum wire and an Ag/AgCl electrode (3.0 M KCl) served as the counter and reference electrodes, respectively. Prior to each measurement, the working electrode was immersed in the test solution for 30 min to stabilize the open circuit potential (OCP). Electrochemical impedance spectroscopy (EIS) was then carried out at the stabilized OCP using a 5 mV AC perturbation over a frequency range of 100 kHz to 0.1 Hz. Impedance data were fitted using an equivalent circuit model to obtain charge transfer resistance (*R*_ct_), solution resistance (*R*_*s*_), and constant phase element (CPE).

The double-layer capacitance (*C*_*dl*_) was calculated from the CPE parameters, and inhibition efficiency (*η %*) was estimated from the variation in *R*_*ct*_. Potentiodynamic polarization (PDP) measurements were carried out within a potential range of ± 300 mV relative to the open-circuit potential (OCP) at a scan rate of 0.5 mV s⁻¹. The corrosion kinetic parameters, including the corrosion current density (*i*_*corr*_), corrosion potential (*E*_*corr*_), polarization resistance (*R*_*p*_), and anodic and cathodic Tafel slopes (*β*_*a*_ and *β*_*c*_), were determined by Tafel extrapolation of the linear portions of the anodic and cathodic branches in the vicinity of *E*_*corr*_ using OrigaMaster 5 software. The corresponding corrosion rates were subsequently calculated from the obtained electrochemical parameters. To guarantee reproducibility, every electrochemical measurement was performed at least three times, and the reported values represent the mean ± standard deviation. The influence of temperature (25–50 °C) and immersion time on inhibition performance was evaluated using EIS under identical conditions.

### Computational details

Computational studies were performed to gain molecular-level insight into the inhibition behavior of the synthesized pyrazole derivatives (**P6** and **P8**) and to support the experimental electrochemical results. Density functional theory (DFT) calculations were carried out using the DMol³ module in BIOVIA Materials Studio v6.0. Calculations were performed in both gas and solvent phases to account for isolated and solution-phase environments. The GGA approach with the BOP functional and DNP 3.5 basis set was employed. Geometry optimizations were conducted without symmetry constraints until convergence was achieved. Frontier molecular orbital energies (*E*_*HOMO*_ and *E*_*LUMO*_) and global reactivity descriptors, including energy gap (*ΔE*), ionization potential (*I*), electronegativity (*χ*), electron affinity (*A*), chemical hardness (*η*), softness (*σ*), and fraction of electron transfer (*ΔN*), were calculated to evaluate the reactivity and interaction tendency of the inhibitors toward the metal surface.

Monte Carlo (MC) simulations were performed using the Adsorption Locator module to investigate adsorption behavior on the Fe (110) surface. A five-layer slab was constructed and expanded into a (25 × 25) supercell. Simulations were carried out in both vacuum and aqueous environments, where the solvent model contained 200 H₂O molecules, 10 H₃O⁺, and 20 Cl⁻ ions to mimic the acidic medium. Each simulation was run for 150,000 steps to ensure equilibrium adsorption configurations.

### Surface morphology examination

The surface morphology of CS and the protective effect of the inhibitor were examined after 10 h of immersion in 1.0 M HCl, both in the absence and presence of the inhibitor at its optimal concentration (5 × 10^–4^ M), using scanning electron microscopy (SEM), energy-dispersive X-ray spectroscopy (EDX), and atomic force microscopy (AFM). SEM observations were carried out using a QUANTA FEG 250 microscope at an accelerating voltage of 20 kV. EDX analysis was employed to study the surface elemental composition, while AFM measurements (SPM-9600, Shimadzu) were used to evaluate surface topography and roughness changes.

## Results and discussion

### Synthesis and structural confirmation of inhibitors

In this work, the condensation of pyrazolyl-thiocarbohydrazone **P1** with 1,3-diphenyl-4-formylpyrazole under reflux conditions in ethyl alcohol and glacial ethanoic acid afforded the bis-pyrazole derivative **P6** (Scheme 1). The formation of **P6** was supported by spectroscopic data, where the FT-IR spectrum revealed the absence of the NH_2_ stretching vibration along with the presence of characteristic NH, C = N, and C = S absorption bands. Furthermore, the ^1^H NMR spectrum displayed two exchangeable singlet signals corresponding to NH protons, as well as distinct singlets assigned to the azomethine (CH = N) and pyrazole C5-H protons, confirming the proposed bis-pyrazole structure (Fig. S1). Similarly, treatment of thiocarbohydrazone **P1** with 3-nitro-ω-bromoacetophenone in refluxing ethyl alcohol including anhydrous sodium acetate, resulted in the formation of the pyrazolyl-thiadiazine derivative **P8** (Scheme 1). The disappearance of the C = S absorption band in the FT-IR spectrum, accompanied by the appearance of NH, C = N, and NO₂ characteristic bands, indicated successful cyclization to the thiadiazine ring. Furthermore, the ^1^H NMR spectrum showed an exchangeable NH proton signal and a singlet corresponding to the methylene (CH_2_) group, while the ^12^C NMR data provided additional confirmation of the thiadiazine framework (Fig. S2). Detailed characterization data and spectra are provided in the Supplementary Information (Section SI (1)).

### Weight loss (WL) measurements

WL measurements clearly demonstrate that both compounds **P6** and **P8** function as effective corrosion inhibitors in 1.0 M HCl for CS surface. In the absence of inhibitors, the corrosion rate (*CR*) increased sharply with immersion time, reflecting the aggressive nature of the acidic medium. The corrosion rate, surface coverage (*θ*), and inhibition efficiency (*η %*) were intended by the standard relationships^24^:

1 $$\:CR=\:\frac{{W}_{o\:}-W}{A\times\:t\:}$$

2 $$\:\theta\:=\:\frac{{W}_{o\:}-W}{{W}_{o}}\:$$

3 $$\:\eta\:=\theta\:\times\:100\:\:\:\:$$

**Fig. 1:**
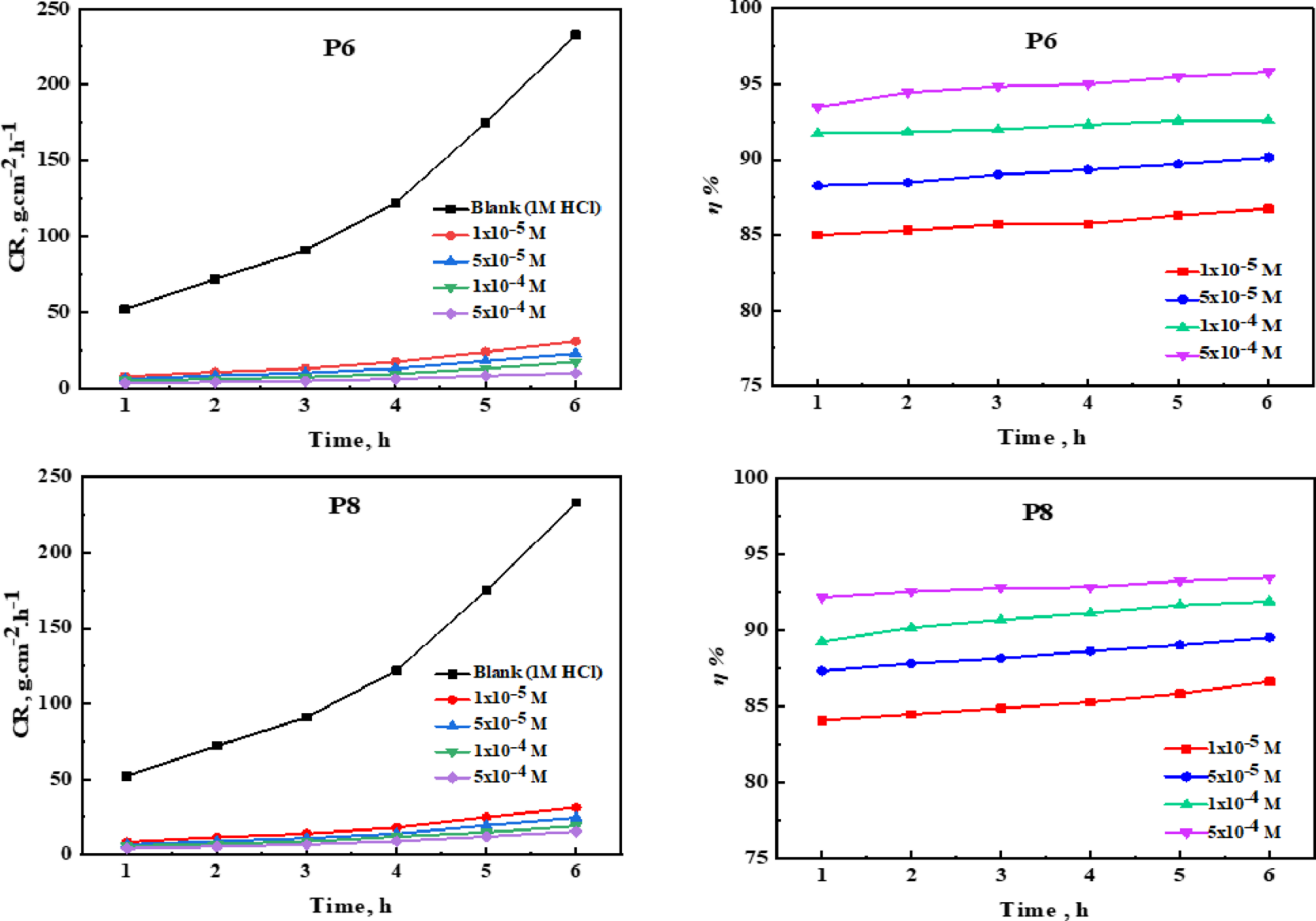
Time-dependent variation of corrosion rate (*CR*) and inhibition efficiency (*η* %) of CS in 1.0 M HCl solution in the presence of various concentrations of **P6** and **P8** inhibitors.

Here, *W*_o_ and *W* denote the weight loss (*g*) of the CS coupons in the absence and presence of the inhibitors, respectively; *A* represents the exposed surface area (cm^2^), and *t* corresponds to the immersion time (*h*). These equations were applied to determine the corrosion behavior under varying concentrations of compounds **P6** and **P8** over immersion periods up to *6 h*. As summarized in Table 1, both compounds significantly reduce the corrosion rate across all tested concentrations. At the highest concentration (5 × 10^–4^ M), compound **P6** achieved a maximum inhibition efficiency of 95.8% after *6 h*, whereas compound **P8** reached 93.4%, highlighting the slightly superior protective action of compound **P6**. The *CR–time* and *η* %–time profiles, Fig. 1, reveal that inhibition efficiency slightly increases with immersion time at lower concentrations, indicating gradual adsorption and self-organization of inhibitor particles on the steel surface^25^.

**Table 1:** Corrosion rate (*CR*) and inhibition efficiency (*η* %) of CS in 1.0 M HCl solution in the absence and presence of **P6** and **P8** inhibitors at different concentrations and immersion times.

Conc,* M*	P6			P8	
	Time (h)	CR ×10^–4^ (g·cm^–2^·h^–1^)	*η %*	CR ×10^–4^ (g·cm^–2^·h^–1^)	*η %*
Blank	1	52	–	52	–
	2	72	–	72	–
	3	91	–	91	–
	4	122	–	122	–
	5	175	–	175	–
	6	233	–	233	–
1 × 10^–5^	1	7.8	85.00	8.3	84.04
	2	10.6	85.30	11.5	84.44
	3	13.0	85.70	13.8	84.84
	4	17.4	85.74	18.0	85.25
	5	24.0	86.30	24.8	85.80
	6	30.9	86.74	31.2	86.61
5 × 10^–5^	1	6.1	88.27	6.6	87.30
	2	8.3	88.47	8.8	87.78
	3	9.98	89.00	10.8	88.13
	4	13.0	89.34	13.9	88.61
	5	18.0	89.70	19.3	89.00
	6	23.0	90.13	24.5	89.50
1 × 10^–4^	1	5.3	91.73	5.6	89.23
	2	5.9	91.81	7.1	90.14
	3	7.3	91.98	8.5	90.66
	4	9.4	92.30	11.9	91.10
	5	13.0	92.57	14.7	91.60
	6	17.3	92.60	18.99	91.85
5 × 10^–4^	1	3.4	93.46	4.1	92.12
	2	4.0	94.44	5.4	92.50
	3	4.7	94.84	6.6	92.75
	4	6.1	95.00	8.77	92.80
	5	8.1	95.48	11.9	93.20
	6	9.8	95.80	15.3	93.43

At advanced concentrations, the efficiency remains nearly constant throughout the immersion period, suggesting that complete surface coverage is rapidly attained, forming a dense, adherent, and stable protective film. The observed increase in *CR* with time at lower inhibitor concentrations is modest compared to the blank, emphasizing the robustness of the adsorbed layer. The superior inhibition performance of compound **P6** can be attributed to its molecular architecture, which contains two pyrazole rings, multiple phenyl groups, and a thiohydrazide moiety, providing numerous adsorption-active sites.

These sites facilitate strong coordination interactions with the vacant d-orbitals of Fe particles and enable π-electron interactions with the metal surface, resulting in a compact and persistent barrier layer. In contrast, compound **P8** contains a pyrazole ring, a thiadiazine ring, and a nitrophenyl group, offering fewer adsorption centers and slightly lower surface coverage, which explains its comparatively lower inhibition efficiency. The existence of heteroatoms (N, S, O), imine (C = N), and aromatic rings in both molecules is crucial for adsorption, film formation, and resistance against acidic attack^26^.

### Electrochemical characterization

#### Electrochemical impedance spectroscopy (EIS)

**Fig. 2:**
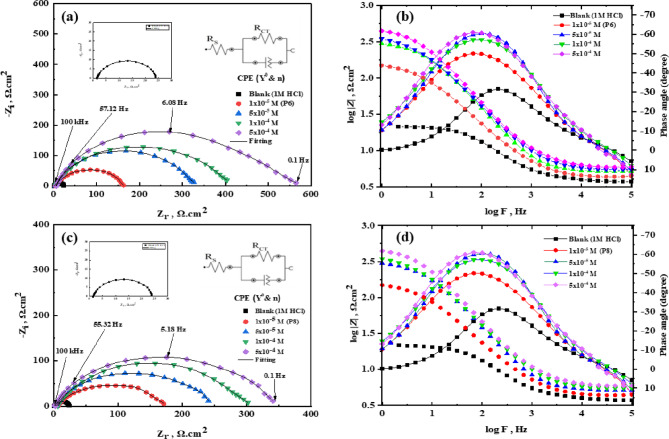
Electrochemical impedance spectra of CS in 1.0 M HCl solution in the absence and presence of **P6** and **P8** inhibitors: Nyquist plots for **P6** (**a**), Bode plots for **P6** (**b**), Nyquist plots for **P8** (**c**), and Bode plots for **P8** (**d**).

The electrochemical impedance response of CS in 1.0 M HCl in the absence and presence of compounds **P6** and **P8** is presented in Fig. 2a–d, with the corresponding parameters listed in Table 2. In the uninhibited acidic medium (blank), the Nyquist plot displays a small and depressed capacitive semicircle with a very limited diameter. This behavior corresponds to a low charge transfer resistance value (*R*_*ct*_ = 22.26 Ω·cm^2^, Table 2), indicating rapid charge transfer at the steel/solution interface^27^. Upon addition of the inhibitors, a significant increase in the diameter of the Nyquist semicircles is observed, particularly at higher concentrations, reflecting enhanced resistance to charge transfer. This is confirmed by the marked increase in *R*_*ct*_ values, which reach 559.8 Ω·cm² for compound **P6** and 335 Ω·cm² for compound **P8** at 5 × 10^–4^ M (Table 2), demonstrating a strong suppression of the corrosion process, which is directly associated with the corrosion reaction rate^28^. Based on *R*_*ct*_ values, the surface coverage (*θ*) and inhibition efficiency (*η* %) were calculated using Eqs. (4) and (5)^29^:

4 $$\:\theta\:=(1-{R}_{ct\left(blank\right)}/{R}_{ct(inh.)})$$

5 $$\:\eta\:\:\%=\theta\:\times\:100$$

**Table 2:** Electrochemical impedance spectroscopy (EIS) parameters for CS in 1.0 M HCl solution in the absence and presence of different concentrations of **P6** and **P8** inhibitors at room temperature.

Inh.	Conc, M	*R*_s_, (Ω.cm^2^)	*R*_ct_, (Ω.cm^2^)	C_dl_, (×10^–6^ F.cm^–2^)	τ (s)	Chi Squared χ^2^ x10^− 3^	*θ*	*η* %
**Blank**	0	1.463 ± 0.082	22.26 ± 0.84	0.886 ± 0.018	0.00447	1.769	–	–
**P6**	1 × 10^−5 ^	4.256 ± 0.121	162.5 ± 1.93	0.736 ± 0.014	0.02325	1.510	0.863	86.3
	5 × 10^−5 ^	5.030 ± 0.146	323.1 ± 2.61	0.818 ± 0.016	0.02269	1.204	0.931	93.11
	1 × 10^− 4^	5.265 ± 0.173	403.1 ± 3.02	0.762 ± 0.015	0.03339	2.134	0.945	94.5
	5 × 10^− 4^	5.808 ± 0.188	559.8 ± 3.78	0.781 ± 0.017	0.04017	1.912	0.960	96.0
**P8**	1 × 10^− 5^	6.702 ± 0.191	161.6 ± 1.88	0.691 ± 0.013	0.02052	1.779	0.862	86.23
	5 × 10^− 5^	4.187 ± 0.137	231.6 ± 2.21	0.752 ± 0.015	0.01770	1.953	0.903	90.34
	1 × 10^− 4^	5.742 ± 0.176	295.3 ± 2.56	0.743 ± 0.014	0.03201	1.411	0.925	92.46
	5 × 10^− 4^	5.830 ± 0.194	335.0 ± 2.87	0.786 ± 0.016	0.02049	2.250	0.934	93.36

Here, $\:{R}_{ct\left(blank\right)}$ and $\:{R}_{ct(inh.)}$ represent the charge transfer resistances of the CS electrode in the absence and presence of the inhibitors (**P6** and **P8**), respectively. All Nyquist plots display a single depressed semicircle without any indication of diffusion-controlled behavior confirming that the corrosion process remains governed by charge transfer in both uninhibited and inhibited systems^30^. The depressed nature of the semicircles is attributed to surface heterogeneity and roughness of the CS electrode^31^. To account for this non-ideal capacitive behavior, a constant phase element (CPE) was employed in place of a pure capacitor in the equivalent circuit model^32^(Fig. 2a and c), whose impedance is described by Eq. (6)^33^:

6 $$\:{Z}_{CPE}={Q}^{-1}(i{\omega\:}_{max}{)}^{-n}$$

Here, *Q* corresponds to the constant phase element, while *ω*_*max*_ represents the angular frequency. The reliability of the fitting procedure was confirmed by the low *χ*^2^ values obtained from the equivalent circuit analysis, indicating excellent agreement between the experimental and simulated impedance data. The low *χ*^2^ values, together with the increase in charge-transfer resistance (*R*_*ct*_) and the decrease in double-layer capacitance (*C*_*dl*_), confirm the formation of a compact and homogeneous protective adsorbed film on the CS surface, which effectively hinders the charge transfer process at the metal/electrolyte interface. Further insight is obtained from the Bode magnitude and phase plots (Fig. 2b and d). According to Eq. (7), the double-layer capacitance (*C*_*dl*_) values were determined from the frequency corresponding to the peak of the maximum imaginary impedance^34^:

7 $$\:{C}_{dl}=1/\left(2\pi\:{R}_{ct}{F}_{img\to\:Max}\right)$$

Here, $\:F$($\:\mathrm{i}\mathrm{m}\mathrm{g}\to\:\mathrm{M}\mathrm{a}\mathrm{x}$), refers to the frequency at which the imaginary component of the impedance reaches its maximum. In addition, the relaxation time constant (*τ*), reflecting the interfacial response rate, was calculated using Eq. (8)^35^:

8 $$\:\tau\:={C}_{dl}\times\:{R}_{ct}$$

The obtained parameters indicate a systematic decrease in *C*_*dl*_ and admittance (*Y*_*₀*_) upon inhibitor addition, accompanied by a shift of the CPE exponent (*n*) toward unity. These trends confirm the progressive adsorption of inhibitor molecules, which replaces water molecules and chloride ions at the steel surface and leads to the formation of a more compact and less heterogeneous interfacial layer^36^. A noticeable increase in *R*_*s*_ was observed after inhibitor addition. This behavior has been attributed to reduced electrolyte conductivity and interfacial modifications caused by adsorption of inhibitor molecules. The adsorption process involves competitive displacement of water molecules and chloride ions by inhibitor species, leading to the formation of a compact protective film on the carbon steel surface and an altered interfacial environment^37^. Moreover, the increase in *τ* values in the presence of inhibitors indicates a slower charge transfer process and the development of a more resistive protective film^38^. A clear distinction is observed between both compounds, where **P6** consistently outperforms **P8**, as evidenced by higher *R*_*ct*_, lower *C*_*dl*_, and longer relaxation times. This behavior can be rationalized in terms of molecular structure.

Compound **P6** possesses a more extended π-conjugated framework and a more favorable electronic distribution, providing multiple adsorption centers that facilitate strong donor–acceptor interactions with vacant d-orbitals of iron. In contrast, the presence of a nitro substituent and a more sterically constrained framework in compound **P8** reduces electron density and limits surface interaction, resulting in comparatively weaker adsorption and lower inhibition efficiency. Finally, the impedance results confirm that both inhibitors significantly enhance corrosion resistance, with compound **P6** forming a more stable, compact, and protective adsorption layer on the CS surface. A comparison table including representative pyrazole-based corrosion inhibitors reported in the literature is presented in Table S2 (Supplementary Information)^15,26,39–44^. The table summarizes the main inhibition performance data of structurally related compounds investigated in hydrochloric acid media and serves to place the present work within the context of previously published studies.

#### Potentiodynamic polarization (PDP)

**Fig. 3:**
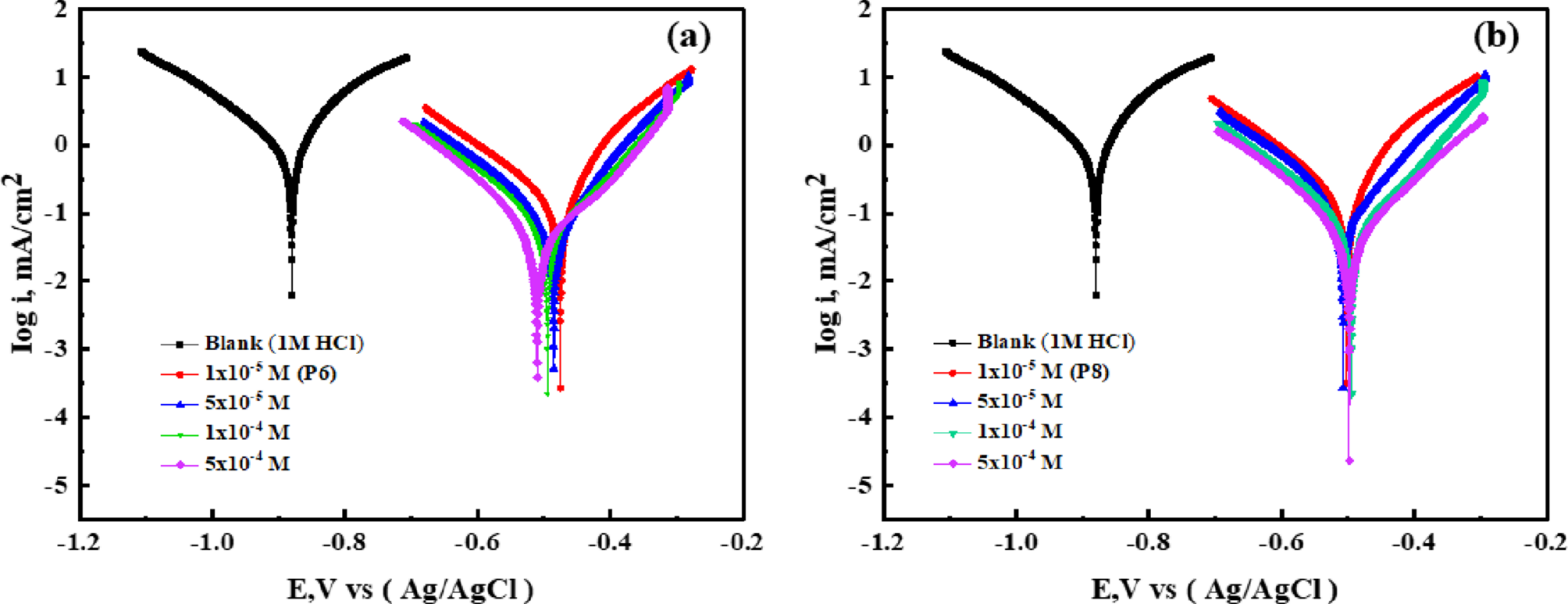
Potentiodynamic polarization curves of CS in 1.0 M HCl solution in the absence and presence of **P6** (**a**) and **P8** (**b**) inhibitors.

The effect of compounds **P6** and **P8** on the anodic and cathodic behavior of carbon steel in 1.0 M HCl was investigated by PDP measurements. The polarization curves are shown in Fig. 3a and b, and the corresponding parameters are listed in Table 3. In the blank solution, CS exhibits a high corrosion current density (*i*_*corr*_ = 627.05 µA·cm⁻²), indicating rapid corrosion kinetics in the aggressive acidic medium, involving both anodic iron dissolution and cathodic hydrogen evolution. This observation is consistent with the low *R*_*ct*_ value obtained from EIS measurements (Table 2), confirming the highly active corrosion state of the steel surface^27^.

**Table 3:** Potentiodynamic polarization parameters for CS in 1.0 M HCl solution in the absence and presence of different concentrations of **P6** and **P8** inhibitors at room temperature.

Inh.	Conc, M	-E_corr_ (mV vs. Ag/AgCl)	β_a_ (mV/dec)	-β_c_ (mV/dec)	i_corr_ µA.cm^− 2^	CR (mm year⁻¹)	*R*_*p*_ (Ω.cm²)	*θ*	*η* %
**Blank**	0	882.3 ± 2.317	60.9 ± 1.284	108.8 ± 1.936	627.05 ± 2.84	14.73	27.1 ± 0.428	–	–
**P6**	1 × 10^− 5^	477.4 ± 1.845	36.1 ± 0.918	50.0 ± 1.227	61.22 ± 1.126	1.44	149 ± 0.736	0.902	90.24
	5 × 10^− 5^	485.6 ± 2.214	56.0 ± 1.362	56.1 ± 1.118	38.48 ± 0.734	0.90	317 ± 1.212	0.939	93.86
	1 × 10^− 4^	495.0 ± 2.687	61.2 ± 1.104	53.1 ± 1.382	32.52 ± 0.618	0.76	380 ± 1.403	0.948	94.80
	5 × 10^− 4^	510.7 ± 2.038	56.0 ± 1.25	50.3 ± 0.964	21.18 ± 0.421	0.50	544 ± 1.276	0.966	96.60
**P8**	1 × 10^− 5^	502.1 ± 1.972	45.5 ± 1.031	60.6 ± 1.527	78.14 ± 1.348	1.84	145 ± 0.682	0.875	87.54
	5 × 10^− 5^	508.5 ± 2.463	70.9 ± 1.714	52.0 ± 1.224	56.64 ± 0.996	1.33	226 ± 0.841	0.910	91.00
	1 × 10^− 4^	494.3 ± 2.146	62.9 ± 1.286	62.9 ± 1.442	46.08 ± 0.827	1.08	297 ± 0.907	0.927	92.65
	5 × 10^− 4^	487.0 ± 1.894	55.9 ± 1.172	58.5 ± 1.363	36.33 ± 0.651	0.85	342 ± 0.965	0.942	94.21

The stability of the CS surface prior to PDP measurements was further confirmed by OCP measurements (Fig. S7, Supplementary Information)^45^. The OCP profiles exhibit gradual stabilization with immersion time, indicating the establishment of a quasi-steady state at the metal/solution interface. In the presence of compounds **P6** and **P8**, a noticeable shift toward more noble potentials compared to the blank solution suggests adsorption of inhibitor molecules and enhanced surface stability. The addition of compounds **P6** and **P8** results in a marked decrease in *i*_corr_, which systematically decreases with increasing inhibitor concentration. At 5 × 10⁻⁴ M, *i*_*corr*_ decreases to 21.18 µA·cm⁻² for **P6** and 36.33 µA·cm⁻² for **P8** (Table 3). Based on these values, the surface coverage (*θ*) and inhibition efficiency (*η* %) were calculated using the following relationships^35^:

9 $$\:\theta\:=({i}_{corr.\:HCl}-{i}_{corr.\:\:inh})/{i}_{corr.\:HCl}$$

10 $$\:\eta\:\:\%=\theta\:\:\times\:\:100$$

Here, $\:{i}_{corr.\:HCl}\:$ and $\:{i}_{corr.\:\:inh}$ correspond to the corrosion current densities in the absence and presence of inhibitors, respectively. At the same time, the polarization resistance (*R*_*p*_) was determined using the Stern–Geary Eq. 4^6^:

11 $$\:\:{R}_{p}={\beta\:}_{a}{\beta\:}_{c}/2.303\:{i}_{corr}\:({\beta\:}_{a}+{\beta\:}_{c}\:)$$

Here, *β*_a_ and *β*_c_ denote the anodic and cathodic Tafel slopes, respectively. The good agreement between the polarization resistance (*R*_*p*_) values obtained from PDP and the charge transfer resistance (*R*_*ct*_) derived from EIS confirms the consistency of the electrochemical results and supports the proposed inhibition mechanism. Although the addition of **P6** and **P8** causes a positive shift in the corrosion potential (*E*_*corr*_) compared to the blank solution (Table 3), this shift does not show a clear concentration-dependent trend^47^.

Both anodic (*β*_a_) and cathodic (*β*_c_) Tafel slopes are affected in the presence of **P6** and **P8**, demonstrating that adsorption of the inhibitor molecules influences the kinetics of both partial electrochemical reactions occurring at the CS surface. The anodic branch of the polarization curves is associated with iron dissolution (Fe → Fe^2+^ + 2e^–^), whereas the cathodic branch corresponds to the hydrogen evolution reaction. The variation in the anodic Tafel slope (*β*_a_) after inhibitor addition suggests that adsorption of **P6** and **P8** modifies the kinetics of the iron dissolution process by blocking active anodic sites and increasing the energy barrier for metal oxidation. Likewise, the changes observed in the cathodic Tafel slope (*β*_c_) indicate that the adsorbed inhibitor layer also affects the hydrogen evolution reaction through partial blockage of cathodic active sites, thereby reducing the rate of proton reduction. The simultaneous modification of both Tafel slopes reflects the influence of the adsorbed inhibitor film on the charge-transfer processes occurring at the metal/solution interface. A substantial positive displacement of *E*_*corr*_ relative to the blank solution was observed for both inhibitors. The magnitude of this displacement exceeds the commonly adopted ± 85 mV criterion, indicating that the overall inhibition behavior is predominantly anodic. Although both anodic and cathodic reactions are influenced by inhibitor adsorption, the much larger positive shift in *E*_*corr*_ suggests that the suppression of the anodic iron dissolution process is the dominant contribution to the overall inhibition mechanism^48,49^. The effect becomes more pronounced with increasing inhibitor concentration, confirming the formation of an adsorbed protective film that effectively retards the corrosion process.

The calculated surface coverage (*θ*) and inhibition efficiency (*η* %) increase with inhibitor concentration, reaching maximum values of 96.6% for **P6** and 94.21% for P8. The higher performance of compound **P6** compared to compound **P8** is attributed to its richer electronic structure and greater availability of adsorption-active centers, which enhance interaction with the steel surface through donor–acceptor mechanisms. This results in a more stable and compact protective film, consistent with its lower *i*_corr_ values and higher polarization resistance. Overall, the polarization results, in agreement with EIS data, confirm the effective inhibition performance of both compounds in acidic media.

### Adsorption and film stability

**Fig. 4:**
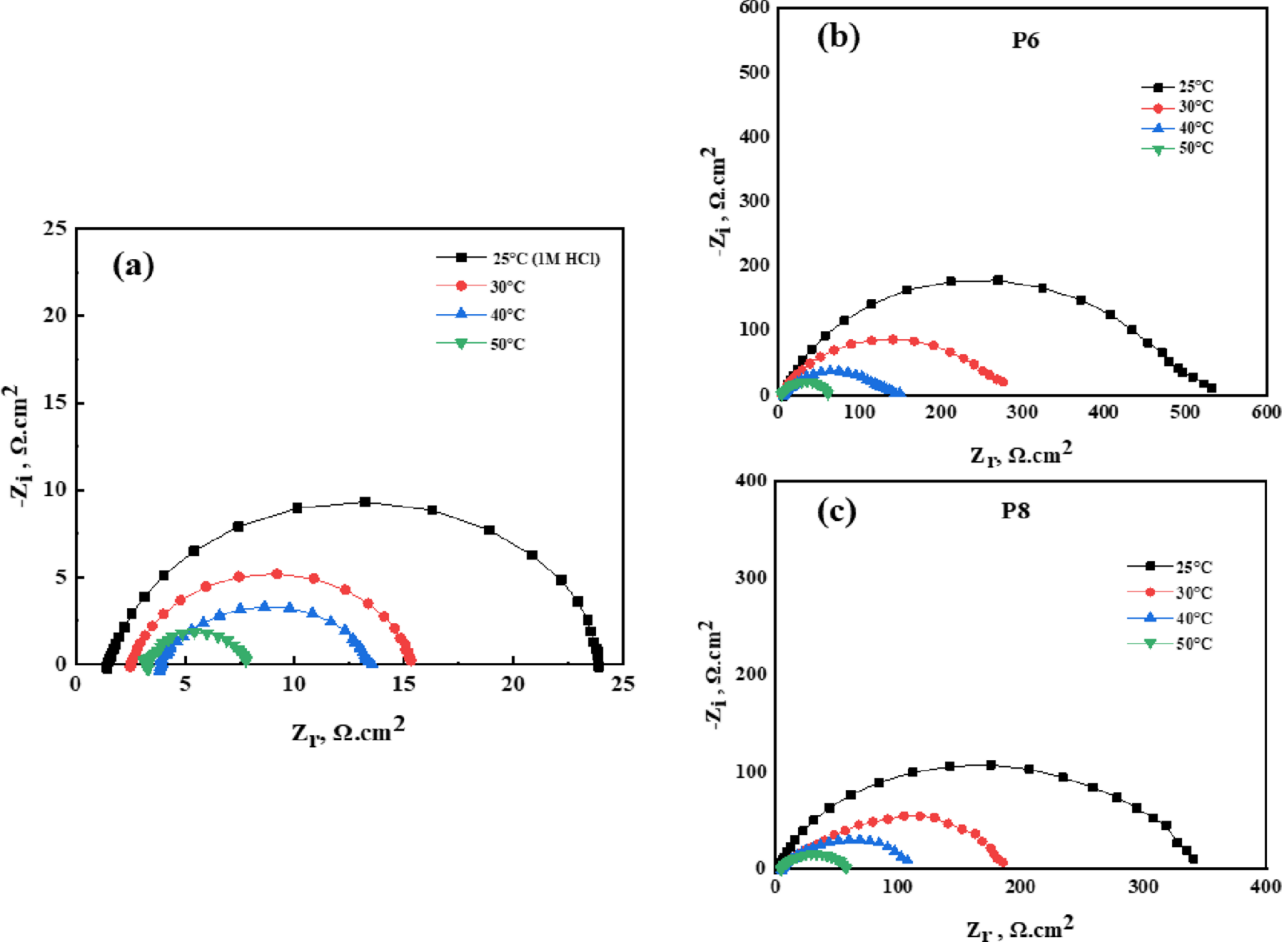
Nyquist impedance plots of CS in 1.0 M HCl solution at different temperatures: blank solution (**a**), in the presence of **P6** (**b**), and in the presence of **P8** (**c**).

The stability of the protective layer formed by compounds **P6** and **P8** on CS in 1.0 M HCl (5 × 10⁻⁴ M) was evaluated under elevated temperatures (25–50 °C) using EIS measurements. The electrochemical parameters are summarized in Table 4. A clear deterioration in the corrosion resistance of the uninhibited system is observed with increasing temperature, as reflected by the sharp decrease in polarization resistance from 22.26 Ω·cm² at 25 °C to 4.77 Ω·cm² at 50 °C. This behavior highlights the highly aggressive nature of the acidic medium and the rapid acceleration of both anodic metal dissolution and cathodic hydrogen evolution in the absence of any protective barrier. In the presence of **P6** and **P8** (5 × 10⁻⁴ M), the Nyquist plots (Fig. 4) retain their characteristic depressed semicircular shape across the investigated temperature range, this observation suggest that the corrosion of CS remains largely controlled by the charge-transfer mechanism^50^.

**Table 4:** Effect of temperature on the electrochemical impedance parameters of CS in 1.0 M HCl solution in the absence and presence of **P6** and **P8** inhibitors.

	*Temp.* °C	*R*_s_, (Ω.cm^2^)	*R*_P_, (Ω.cm^2^)	*θ*	*η %*
**Blank**	25	1.463	22.26	–	–
	30	2.50	13.03	–	–
	40	3.879	9.39	–	–
	50	3.111	4.77	–	–
**P6**	25	5.808	559.80	0.96	96
	30	3.696	286.90	0.9546	95.46
	40	5.701	142.20	0.934	93.40
	50	4.284	58.79	0.9208	92.08
**P8**	25	5.830	335.0	0.9336	93.36
	30	4.486	179.90	0.9276	92.76
	40	5.081	105.30	0.9101	91.01
	50	4.554	52.14	0.9085	90.85

However, a gradual reduction in semicircle diameter is evident as the temperature increases, signifying a decrease in charge-transfer resistance. For compound **P6**, *R*_*p*_ decreases from 559.8 Ω·cm^2^ at 25 °C to 58.79 Ω·cm^2^ at 50 °C, while for P8 it decreases from 335 Ω·cm^2^ to 52.14 Ω·cm^2^ over the same temperature interval (Table 4). Despite this reduction, the *R*_*p*_ values in the inhibited systems remain meaningfully higher than those of the blank solution at all temperatures, confirming the persistent protective action of both inhibitors under thermal stress. The corresponding inhibition efficiencies show only a moderate decline with temperature, reaching 92.08% for compound **P6** and 90.85% for compound **P8** at 50 °C.

This behavior suggests that although partial desorption of inhibitor molecules may occur at elevated temperatures, a substantial fraction of the adsorbed film remains intact and continues to block active corrosion sites^51,52^. These features promote stronger adsorption and improved resistance of the protective film against thermal agitation, allowing compound **P6** to maintain a more effective barrier even at elevated temperatures. The effect of immersion time on the stability of protective films was also investigated, and the results are provided in Supporting Information (Section SI (2), Fig. S3, Table S1).

### Activation thermodynamic parameters

**Fig. 5:**
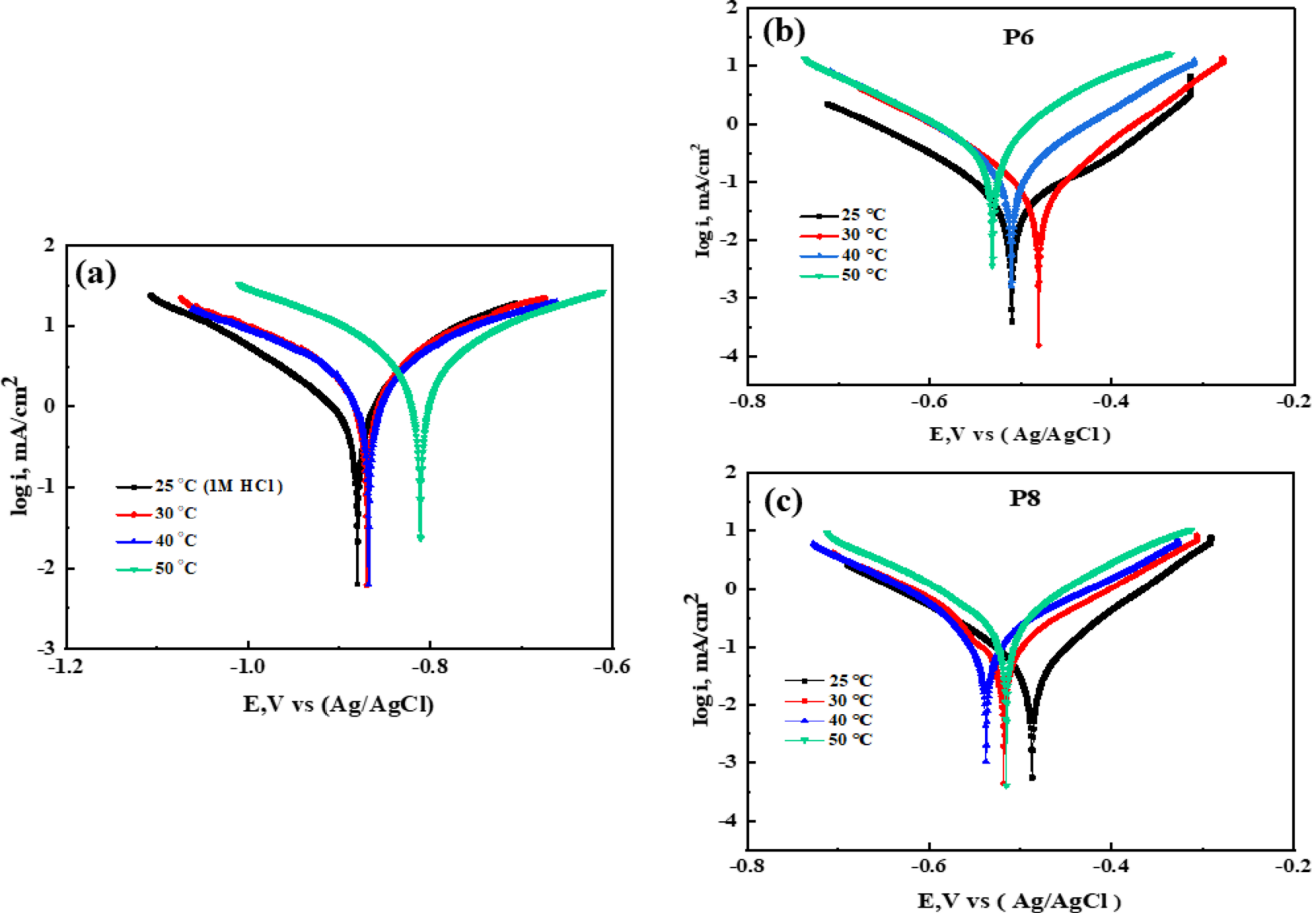
Potentiodynamic polarization curves of CS in 1.0 M HCl solution at different temperatures: blank solution (**a**), in the presence of **P6** (**b**), and in the presence of **P8** (**c**).

**Fig. 6:**
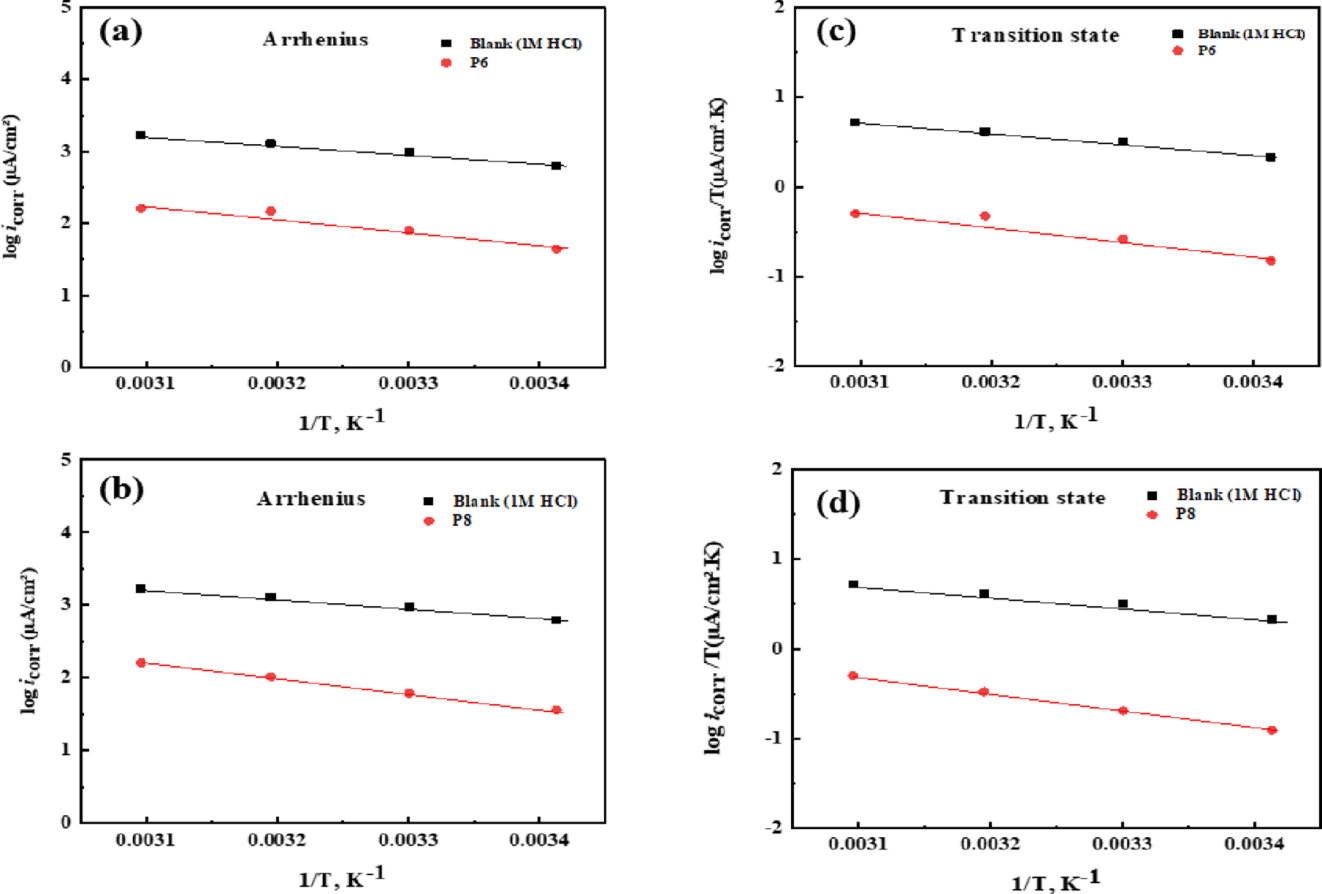
Arrhenius plots of log *i*_*corr*_ versus 1/T for CS in 1.0 M HCl solution in the presence of **P6** (**a**) and **P8** (**b**), and corresponding transition-state plots of log (*i*_*corr*_ /T) versus 1/T for **P6** (**c**) and **P8 **(**d**).

To further elucidate the temperature-dependent inhibition behavior of compounds **P6** and **P8**, PDP measurements were performed at different temperatures using a concentration of 5 × 10⁻⁴ M. The polarization curves (Fig. 5a–c) and the corresponding parameters (Table 5) provide insight into the corrosion kinetics of CS in both uninhibited and inhibited solutions. In the absence of inhibitors, *i*_*corr*_ increased markedly from 627.05 to 1682.57 µA.cm⁻² with increasing temperature (25–50 °C), reflecting the accelerated dissolution of carbon steel in the acidic medium. In contrast, although *i*_*corr*_ of the inhibited systems also increases with temperature, they remain significantly lower than those of the blank over the entire temperature range. At 50 °C, *i*_*corr*_ reaches 148.23 µA.cm^–2^ for compound **P6** and 162.19 µA.cm^–2^ for compound **P8**, confirming the persistence of their protective effect even under thermal conditions. This behavior indicates that the adsorbed inhibitor layers are not completely destabilized by temperature and continue to suppress the corrosion process. The corrosion current density values listed in Table 5 were further used to construct Arrhenius and transition-state plots (Fig. 6a–d) according to the following relations ^45,53^:

12 $$\text{Arrhenius: }{ln}\left({i}_{corr}\right)={ln}A-\left(\frac{{E}_{a}}{RT}\right)$$

13 $$\text{Transition state: }{ln}({i}_{corr}/T)=\left[{ln}\left(\frac{R}{{N}_{A}h}\right)+\left(\frac{\varDelta\:{S}^{*}}{R}\right)\right]-(\varDelta\:{H}^{*}/RT)$$

**Table 5:** Activation thermodynamic parameters for CS corrosion in 1.0 M HCl solution in the absence and presence of **P6** and **P8** inhibitors.

Inh.	Temp. °C	i_corr_ µA.cm^− 2^	1 / T	log (i_corr_)	log (i_corr_ /T)	Slope (Arrhenius)	Slope (transition-state)	Intercept (transition-state)	∆E_a_, kJ.mol^–1^	∆H*, kJ.mol^–1^	∆S*, J.mol^− 1^.K^− 1^
Blank	25	627.051	0.0034	2.8	0.33	–1340.9	–1207.3	4.4661	29.68	27.12	–99.58
	30	962.389	0.0033	2.98	0.5						
	40	1289.57	0.0032	3.11	0.61						
	50	1682.57	0.0031	3.23	0.72						
P6	25	21.179	0.0034	1.33	–1.14	–2643.7	–2510.2	7.4326	50.62	48.07	–55.27
	30	44.013	0.0033	1.64	–0.84						
	40	79.537	0.0032	1.9	–0.59						
	50	148.23	0.0031	2.17	–0.34						
P8	25	36.3344	0.0034	1.56	–0.91	–2061	–1927.4	5.6727	39.46	36.91	–88.96
	30	61.749	0.0033	1.79	–0.69						
	40	104.26	0.0032	2.02	–0.48						
	50	162.19	0.0031	2.21	–0.3						

In this equation, *A* represents the Arrhenius constant, *T* is the absolute temperature, and *R* denotes the universal gas constant. The Arrhenius plots of *ln i*_*corr*_ *versus 1/T* (Fig. 6a, b) show good linearity for both uninhibited and inhibited systems, confirming Arrhenius-type behavior. The calculated activation energy values (Table 5) reveal a clear increase upon inhibitor addition, rising from 29.68 kJ·mol^–1^ for the blank solution to 50.62 kJ·mol^–1^ for compound **P6** and 39.46 kJ·mol^–1^ for compound **P8**. This increase indicates the formation of an energy barrier that hinders the corrosion process^6,54^. This behavior is characteristic of an inhibition process controlled by surface blocking, where adsorbed inhibitor molecules reduce the number of active sites available for charge transfer^55^. The higher increase in *E*_a_ for **P6** compared to **P8** further explains its superior inhibition efficiency, reflecting a stronger kinetic barrier to corrosion.

Additional insight was obtained from the linear transition-state plots of *ln (i*_*corr*_ */T) versus 1/T* (Fig. 6c, d), which enabled the calculation of ΔH^*^ and ΔS^*^ (Table 5). The positive ΔH^*^ values in all cases confirm the endothermic nature of the dissolution process. Notably, ΔH^*^ increases from 27.12 kJ·mol⁻¹ for the blank to 48.07 kJ·mol^–1^ for compound **P6** and 36.91 kJ·mol⁻¹ for compound **P8**, indicating that higher energy is required for corrosion in the presence of inhibitors due to the formation of a protective adsorbed layer. ^54,56^. The negative ΔS^*^ values for all systems indicate the formation of a more ordered activated complex compared to the reactants. In the presence of inhibitors, the less negative ΔS^*^ values, particularly for compound **P6** (− 55.27 J·mol^–1^·K^–1^) compared to the blank (− 99.58 J·mol^–1^·K^–1^), suggest a more organized transition state, where the formation of the activated complex is favored over dissociation^54^. Overall, the kinetic and thermodynamic analyses confirm that compounds **P6** and **P8** inhibit corrosion *via* adsorption, with **P6** showing superior performance.

### Adsorption isotherm

**Fig. 7:**
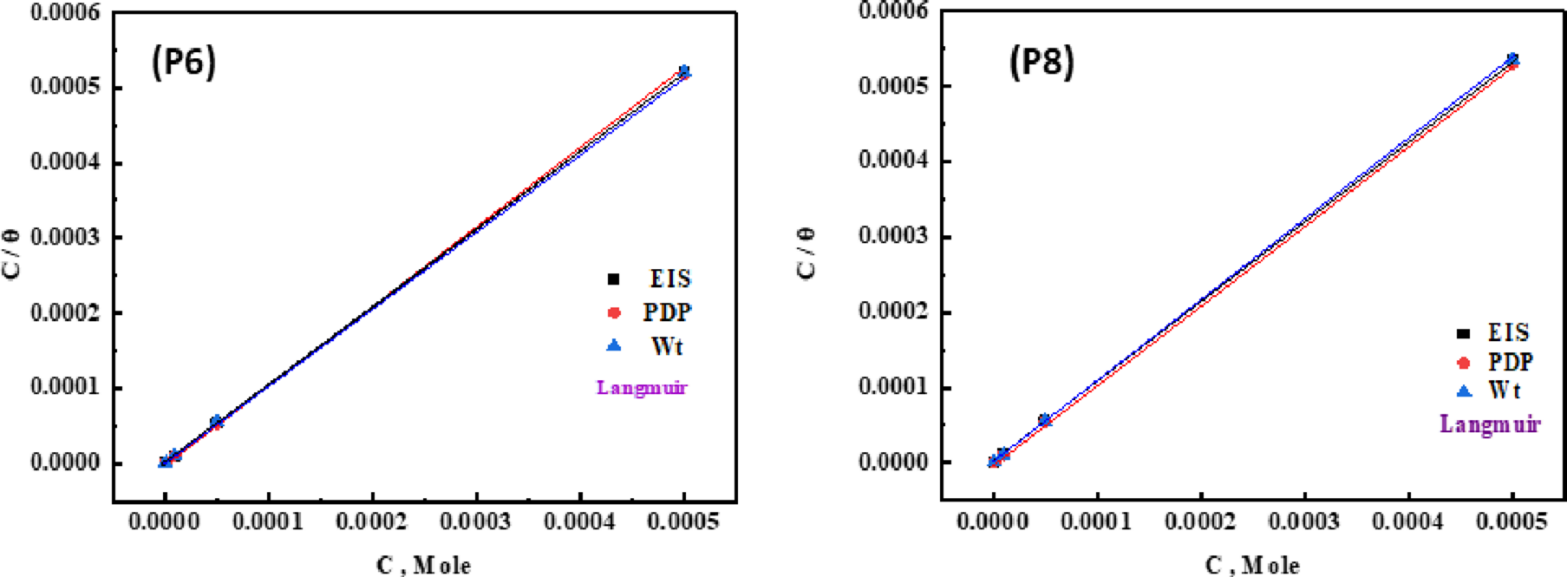
Adsorption isotherm plots describing the adsorption behavior of **P6** and **P8** inhibitors on the CS surface in 1.0 M HCl solution.

The adsorption behavior of pyrazole-based inhibitors **P6** and **P8** on the CS surface in 1.0 M HCl was evaluated using different adsorption isotherm models based on the surface coverage (*θ*) values obtained from PDP, EIS, and weight loss measurements^57^. The corresponding adsorption plots are presented in Fig. 7, while the calculated parameters are summarized in Table 6. Among the tested models, the Langmuir isotherm (Fig. 7) provided the best fit to the experimental data, as indicated by slopes close to unity and correlation coefficients (*R²*) approaching unity. Other isotherm models are included in the Supplementary Information (Figs. S4 and S5) for comparison. This behavior suggests that the adsorption of compounds **P6** and **P8** occurs through the formation of a monolayer on a relatively homogeneous surface, with negligible lateral interaction between the adsorbed species. The Langmuir adsorption isotherm can be expressed as follows^58^:

**Table 6:** Adsorption parameters of **P6** and **P8** inhibitors on the CS surface in 1.0 M HCl solution obtained from PDP, EIS, and weight loss measurements.

Inh.	Method	Slope	*R* ^2^	Log K_ads_ (L.mol⁻¹)	−ΔG°_ads_ (kJ.mol^–1^)
**P6**	PDP	1.028	0.997	6.412	46.873
	EIS	1.041	0.998	6.536	47.621
	Wt.	1.019	0.995	6.287	45.982
**P8**	PDP	1.053	0.996	6.214	45.411
	EIS	1.067	0.997	6.338	46.162
	Wt.	1.031	0.994	6.082	44.873

14 $$\:\raisebox{1ex}{$C$}\!\left/\:\!\raisebox{-1ex}{$\theta\:$}\right.=\left(\frac{1}{{K}_{ads}}\right)+C$$

Here, *C* denotes the inhibitor concentration (mol·L^–1^), *θ* represents the fractional surface coverage, and *K*_*ads*_ is the adsorption equilibrium constant^59^. The relatively high *K*_*ads*_ values indicate a strong affinity of both inhibitors toward the carbon steel surface, reflecting an efficient adsorption process. Notably, compound **P6** exhibits a higher *K*_*ads*_ value than **P8**, signifying stronger adsorption and explaining its superior inhibition efficiency observed in both PDP and EIS measurements. The standard Gibbs free energy of adsorption (*ΔG°*_*ads*_) was calculated according to the thermodynamic equation below^60^:

15 $$\:\varDelta\:{G}_{ads}^{^\circ\:\:}=-RT{ln}\left(55.5{K}_{ads}\right)$$

Here, *R* is the universal gas constant, *T* represents the absolute temperature, and the constant 55.5 corresponds to the molar concentration of water in the solution^59^. For compounds **P6** and **P8**, the − Δ*G*°_*ads*_ values were determined to range from 44 to 46 kJ·mol^–1^, indicating a spontaneous adsorption process and confirming the strong interaction of the inhibitor molecules with the carbon steel surface^61^. It should be noted that although Δ*G*°_*ads*_ is widely used to evaluate adsorption strength, recent studies have emphasized that it cannot, by itself, distinguish between physical and chemical adsorption mechanisms, as corrosion inhibition involves a combination of electrostatic interactions and specific chemical interactions^62,63^. In this context, the adsorption of compounds **P6** and **P8** is better described as a mixed adsorption process rather than a purely single-type mechanism.

Initial adsorption may occur through electrostatic interactions between the protonated inhibitor species and the charged metal surface in acidic media, followed by additional stabilization through donor–acceptor interactions^64^. These interactions involve the lone pair electrons of heteroatoms (N, S, O) and π-electrons of aromatic systems with the vacant 3d orbitals of iron, leading to the formation of a stable protective film^65^. The superior performance of compound **P6** compared to **P8** is related to its higher density of adsorption-active centers and more favorable electronic distribution, which promote stronger surface interaction and the formation of a more compact adsorbed layer.

Overall, the adsorption isotherm results are consistent with the electrochemical findings, indicating strong adsorption behavior of both inhibitors, with compound **P6** exhibiting higher efficiency.

### Quantum chemical calculation

**Fig. 8:**
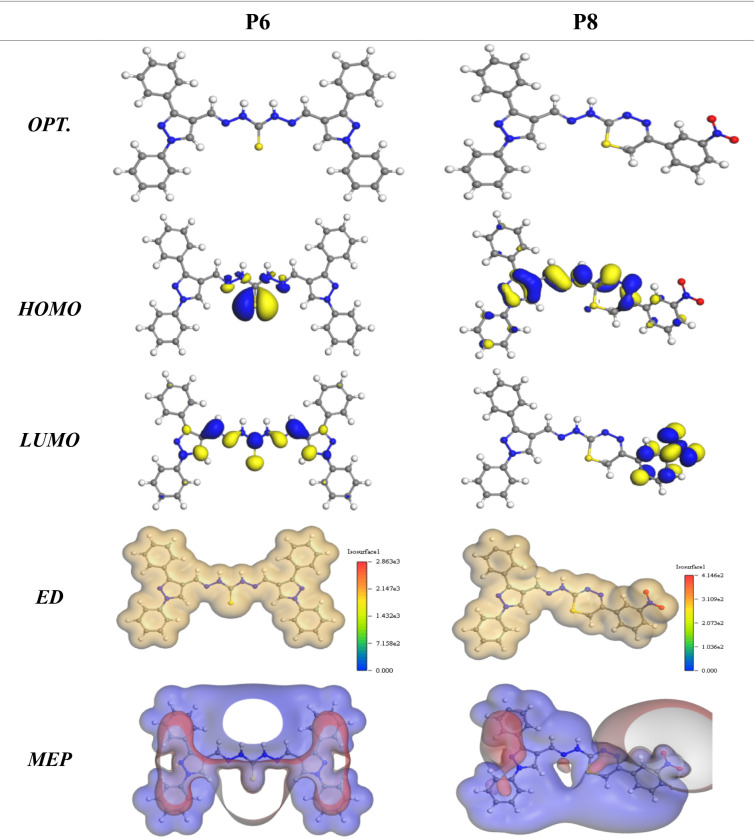
Optimized structures, HOMO, LUMO, ED, and MEP of the studied **P6** and **P8** inhibitors in solvent phase.

The optimized molecular structures, frontier molecular orbitals (HOMO–LUMO), molecular electrostatic potential (MEP) maps, and electron density distributions of compounds **P6** and **P8** in the solvent phase are presented in Fig. 8. The corresponding gas-phase results are provided in the Supporting Information (Fig. S6) for comparison. Quantum chemical parameters, including *E*_*HOMO*_, *E*_*LUMO*_, *ΔE*, electron affinity (*A*), ionization potential (*I*), softness (*σ*), chemical hardness (*η*), and fraction of electron transfer (*ΔN*), were calculated as per the following equations and summarized in Table 7^24^.

**Table 7:** Quantum chemical parameters of **P6** and **P8** inhibitors calculated using density functional theory in gas and solvent phases.

Inh.	Phase	E_HOMO_ (eV)	E_LUMO_ (eV)	Δ E (eV)	A (eV)	I (eV)	X (eV)	*η* (eV)	s	ΔN
**P6**	Gas	-4.740	-3.088	1.651	3.088	4.740	3.914	0.825	1.211	1.86
	Solvent	-4.781	-3.540	1.240	3.540	4.781	4.160	0.620	1.612	2.28
**P8**	Gas	-4.627	-2.438	2.190	2.438	4.627	3.532	1.094	0.914	1.58
	Solvent	-4.205	-2.222	1.982	2.222	4.205	3.214	0.991	1.009	1.91

16 $$\:\varDelta\:{E}_{gap}={E}_{LUMO}-{E}_{HOMO}$$

17 $$\:A=-{E}_{LUMO}$$

18 $$\:I=-{E}_{HOMO\:\:}$$

19 $$\:\eta\:=\frac{\varDelta\:{E}_{gap}}{2}$$

20 $$\:\sigma\:=\frac{1}{\eta\:}$$

21 $$\:\varDelta\:N=\frac{{X}_{Fe}-{\:X}_{inh}}{2\left({\eta\:}_{Fe}+\:{\eta\:}_{inh}\right)}$$

Geometry optimization indicates that both inhibitors adopt nearly planar conformations, enhancing surface coverage and facilitating adsorption on the iron surface. Frontier molecular orbital analysis further provides insight into their reactivity and inhibition tendency. As listed in Table 7, both compounds **P6** and **P8** exhibit relatively high *E*_*HOMO*_ values, indicating their strong ability to donate electrons to the vacant d-orbitals of surface iron atoms. This electron-donating capability supports the formation of coordination bonds and explains the experimentally observed reduction in corrosion current density and increase in charge-transfer resistance upon inhibitor addition^66^.

A clear distinction between the two inhibitors arises from their HOMO–LUMO energy gaps (*ΔE*). Compound **P6** exhibits significantly lower *ΔE* values than **P8** in both the gas phase (1.651 eV for **P6** vs. 2.190 eV for **P8**) and solvent phase (1.240 eV for **P6** vs. 1.982 eV for **P8**). The lower *ΔE* of compound **P6** reflects higher molecular softness and enhanced chemical reactivity, which facilitates electron polarization and strengthens interfacial interactions with the metal surface. The further reduction of *ΔE* upon solvation highlights the stabilizing role of the aqueous acidic medium, which promotes charge delocalization and increases the reactivity of the inhibitor molecules under realistic corrosion conditions^67^.

Additional quantum descriptors reinforce this trend. Compound **P6** displays lower chemical hardness (*η*) and higher softness (*σ*) values compared to **P8**, indicating a greater tendency to undergo electronic deformation during adsorption. Moreover, the fraction of electron transfer (*ΔN*) for **P6** is higher in both gas and solvent phases, confirming its superior capability to transfer electrons to the iron surface. This enhanced charge-transfer capability directly correlates with the higher *θ*, increased *R*_*ct*_, and decreased *C*_*dl*_ observed experimentally, as the formation of a compact adsorbed layer reduces the dielectric constant at the metal/solution interface. The spatial distribution of the frontier orbitals further clarifies the adsorption mechanism. HOMO density in both inhibitors is mainly localized over azomethine (C = N) groups and nitrogen-containing aromatic rings, indicating that these sites act as the primary electron-donating centers responsible for interaction with the vacant d-orbitals of iron. This electron donation initiates the adsorption process and facilitates the formation of a stable coordination bond between the inhibitor molecules and the metal surface^68^.

In contrast, the LUMO distributions are spread over π-conjugated systems and heteroatom-containing moieties, suggesting the ability of the inhibitor molecules to accept electron density from the filled d-orbitals of iron. This back-donation interaction further stabilizes the adsorbed layer and enhances the overall adsorption strength, contributing to the formation of a compact protective film^69^. The MEP maps (Fig. 8) reveal pronounced negative potential regions around nitrogen and oxygen atoms, particularly for compound **P6**, indicating strong electrostatic attraction toward positively charged iron sites^24^. Overall, the frontier molecular orbital analysis confirms that the corrosion inhibition mechanism is governed by a mixed adsorption process involving both electrostatic interactions and donor–acceptor electron transfer between the inhibitor molecules and the iron surface, which is consistent with the experimental observations.

### Monte Carlo adsorption simulation

**Fig. 9:**
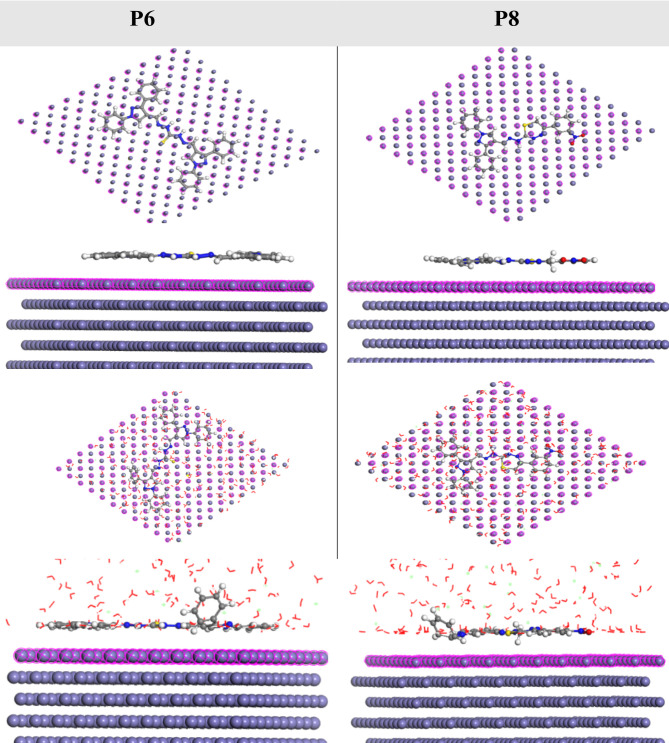
Equilibrium adsorption configuration of the studied **P6** and **P8** inhibitors in both gas and solvent phases on the Fe(110) obtained by MC simulation at room temperature.

MCs were performed to analyze the adsorption behavior of compounds **P6** and **P8** on the Fe (110) surface, and the most stable configurations are revealed in Fig. 9 with the corresponding energetic parameters listed in Table 8. Both inhibitors adsorb preferentially in a nearly parallel orientation, maximizing surface coverage and interaction with the metal surface^70,71^. In the gas phase, compound **P8** exhibits slightly more negative adsorption energy (− 378.9 kJ·mol⁻¹) than **P6** (− 336.8 kJ·mol⁻¹), indicating stronger intrinsic adsorption under isolated conditions. However, in the simulated aqueous environment containing H₂O, H₃O⁺, and Cl⁻ ions, **P6** shows markedly more negative adsorption energy and higher deformation energy (*E*_*def*_ = − 125.9 kJ·mol⁻¹) compared to P8 (*E*_*def*_ = − 8.7 kJ·mol⁻¹), reflecting greater molecular flexibility and stronger surface interaction under realistic corrosive conditions. This enhanced adaptability enables **P6** to effectively displace water molecules and chloride ions from the Fe surface, resulting in a more compact and stable protective film. The stronger adsorption stability of **P6** in the solvated system explains its higher *η* %, increased *R*_*ct*_, and reduced *I*_*corr*_ observed experimentally, demonstrating good agreement between the computational and electrochemical results.

**Table 8:** Monte Carlo simulation parameters for the adsorption of **P6** and **P8** inhibitors on Fe (110) surface in gas phase and simulated acidic solution.

Inh.	Phase	E_T_ (kJ. mol⁻¹)	E_ads_ (kJ. mol⁻¹)	E_rig.ads_ (kJ. mol⁻¹)	E_def._ (kJ. mol⁻¹)	3D Atomistic	H_2_O	H_3_O^+^	Cl^−^
P6	Gas	54.10	–336.75	–259.07	–77.68	–336.75	–	–	–
	Solvent	–5553.80	–6142.47	–6016.56	–125.91	–405.53	–15.90	–164.06	–137.27
P8	Gas	–27.03	–378.91	–327.90	–51.01	–378.91	–	–	–
	Solvent	–4451.26	–4676.62	–4667.90	–8.72	–368.87	–14.46	–164.21	–136.84

### Surface analysis (SEM, EDX, and AFM)

**Fig. 10:**
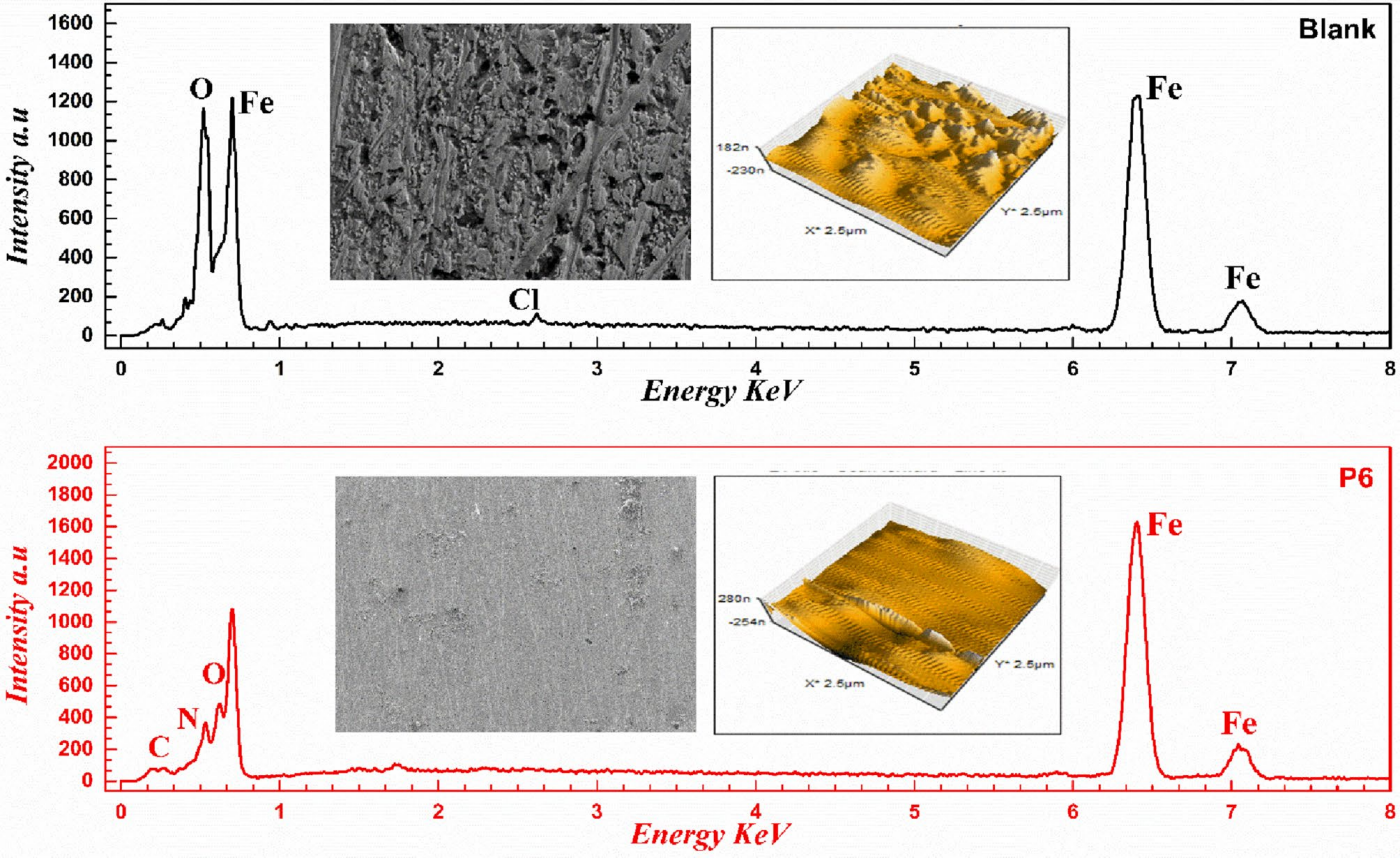
SEM, EDX, and AFM analyses of CS surfaces immersed in 1.0 M HCl without and with P6 (5 × 10^− 4^ M).

SEM micrographs clearly show severe surface deterioration of carbon steel after immersion in 1.0 M HCl, with extensive pits and corrosion defects observed in the absence of **P6** (Fig. 10). In contrast, the presence of P6 (5 × 10⁻⁴ M) results in a significantly smoother surface with markedly reduced damage, indicating effective surface protection through inhibitor adsorption. EDX analysis confirms these observations, where the blank sample shows dominant Fe, O, and Cl signals corresponding to corrosion products, while the inhibited surface exhibits additional C and N signals from the adsorbed **P6** layer, along with a strong reduction in Cl content, indicating displacement of aggressive ions^72,73^.

**Fig. 11:**
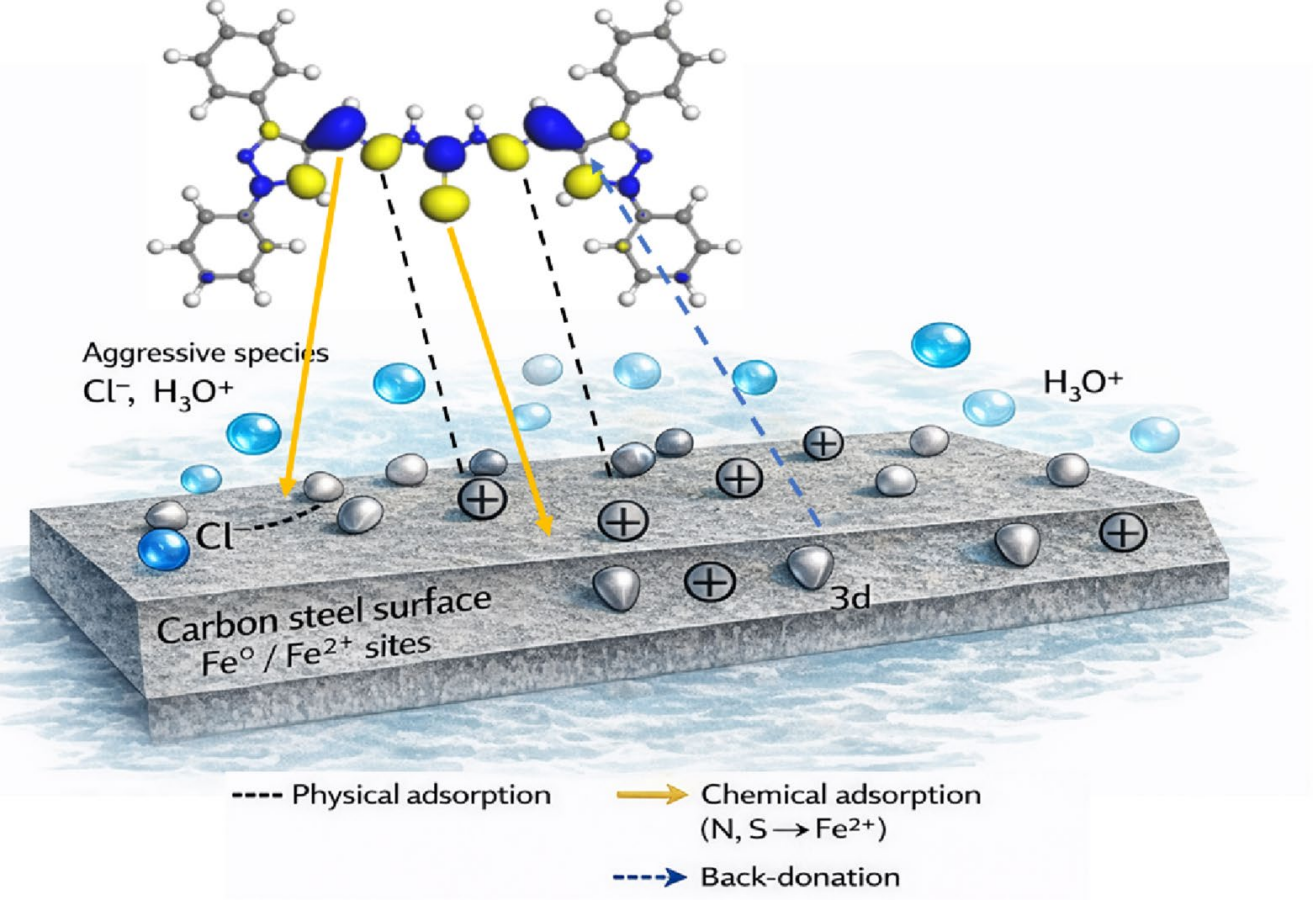
Schematic illustration of the inhibition mechanism of **P6** on the CS surface.

AFM measurements further quantify this improvement, where the average roughness (*Ra*) decreases from 35.27 nm (blank) to 16.53 nm in the presence of compound **P6**, confirming the formation of a compact and uniform protective film^74^. Overall, these results demonstrate that compound **P6** forms an effective adsorbed layer that isolates the steel surface from the corrosive medium. This behavior is consistent with the proposed inhibition mechanism, where adsorption occurs through mixed interactions involving electrostatic attraction and coordination between heteroatoms and iron atoms, as illustrated in Fig. 11. The surface analysis results are in excellent agreement with the theoretical predictions and electrochemical findings.

## Conclusion

This study provided a comprehensive experimental and theoretical evaluation of two newly synthesized pyrazole-based corrosion inhibitors, **P6** and **P8**, for CS in 1.0 M HCl solution. The direct comparison between the two molecular architectures under identical conditions revealed a clear structure–activity relationship, where the bis-pyrazole derivative (**P6**) exhibited superior inhibition performance compared to the pyrazolyl-thiadiazine derivative (**P8**). Electrochemical measurements confirmed a substantial reduction in corrosion activity through the formation of an adsorbed protective film that effectively suppressed both anodic and cathodic reactions, with a more pronounced influence on the anodic dissolution process. Temperature, thermodynamic, and adsorption studies demonstrated the strong affinity of both inhibitors toward the steel surface and indicated that corrosion protection is governed by a mixed adsorption mechanism involving electrostatic interactions and donor–acceptor electron transfer. Furthermore, DFT calculations and Monte Carlo simulations provided molecular-level insight into the inhibition process and confirmed the stronger adsorption tendency and higher reactivity of **P6** toward the Fe (110) surface. Overall, this work provides valuable insight into the structure–activity relationship of pyrazole-based inhibitors and supports the rational design of efficient corrosion protection systems.

## Supplementary Information

Below is the link to the electronic supplementary material.

Supplementary Material 1

## Data Availability

All data generated or analyzed during this study are included in this published article and its supplementary information files.
