# Supplementary material for: Structure-activity relationship of pyrazole-based corrosion inhibitors for carbon steel in HCl: combined experimental and theoretical study
Source: Sci Rep. 2026 Jul 19;16:22556. doi: 10.1038/s41598-026-61895-8 (PMC13381538; doi:10.1038/s41598-026-61895-8)
Supplement: Supplementary file 1 — Supplementary Material 1 [file 41598_2026_61895_MOESM1_ESM.docx]

**Supplementary information:**

**SI (1). Synthesis and characterization of P6 and P8**

Melting points were measured on a MEL-TEMP II electrothermal apparatus and are presented as uncorrected values. Elemental composition (C, H, and N) was determined using a PerkinElmer 2400 CHN analyzer (Perkin-Elmer, Waltham, MA) at the Faculty of Science, Ain Shams University. FT-IR spectra (ν, cm⁻¹) were acquired in KBr discs on a Thermo Scientific Nicolet iS10 spectrometer (Thermo Fisher Scientific Inc., Waltham, MA) at the same institution. The ¹H and ¹³C NMR spectra (δ, ppm) were recorded at 400 and 100 *MHz*, respectively, on a Bruker spectrometer at the Faculty of Pharmacy, Ain Shams University, using DMSO-d₆ as solvent and tetramethylsilane as the internal reference. Reaction monitoring and purity assessment were conducted by thin-layer chromatography on silica gel F_254_ aluminum plates.

***Synthesis of bis-pyrazole derivative (P6)***

The bis-pyrazole derivative, 2-((1,3-diphenylpyrazole-4-yl)methylene)-N′-((1,3-diphenylpyrazol-4-yl)methylene)hydrazine-1-carbothiohydrazide (designated as **P6**) was prepared through a condensation reaction between pyrazolyl-thiocarbohydrazone **P1** (0.01 mol) and 1,3-diphenyl-4-formylpyrazole (0.01 mol). The reaction mixture was refluxed in 20 mL of absolute ethanol with 1 mL of glacial acetic acid for 3 h. Upon cooling, the resulting solid was filtered, thoroughly washed, and recrystallized from dioxane, yielding **P6** as orange crystals in 81% yield, with a melting point of 245–247 °C. FT-IR (KBr, ν, cm⁻¹): absorption bands were observed at 3339 and 3185 (NH stretching), 1598 (C=N stretching), and 1242 (C=S stretching). ¹H NMR (400 MHz, DMSO-d₆, δ, ppm): signals appeared at 7.38 (t, 2H, aromatic H, J = 7.2 Hz), 7.42–7.60 (m, 10H, aromatic H), 7.73 (d, 4H, aromatic H, J = 7.2 Hz), 7.90 (d, 4H, aromatic H, J = 8.0 Hz), 8.29 (s, 2H, CH=N), 9.25 (s, 2H, pyrazole C5-H), 10.11 (broad s, 1H, NH), and 11.78 (broad s, 1H, NH). Elemental analysis for C₃₃H₂₆N₈S (M = 566.69 g/mol): calculated values: C, 69.94%; H, 4.62%; N, 19.77%. Experimental results: C, 69.82%; H, 4.57%; N, 19.75%.

**Synthesis of pyrazolyl-thiadiazine derivative (P8)**

The pyrazolyl-thiadiazine derivative, 2-(2-((1,3-diphenyl-1H-pyrazol-4-yl)methylene) hydrazinyl)-5-(3-nitrophenyl)-6H-1,3,4-thiadiazine (abbreviated as **P8**), was obtained *via* cyclocondensation of pyrazolyl-thiocarbohydrazone **1** (0.01 mol) with 3-nitro-ω-bromoacetophenone (0.01 mol). The reaction was refluxed in 15 mL of ethanol with 0.013 mol of anhydrous sodium acetate for 4 h. Upon completion, the formed solid was collected by filtration, washed with ethanol, and recrystallized, affording **P8** as a crystalline product in 73% yield with a melting point of 162–164 °C. FT-IR (KBr, ν, cm⁻¹) showed characteristic absorptions at 3200 (NH), 1600 (C=N), and 1528, 1348 (NO₂). ¹H NMR (400 MHz, DMSO-d₆, δ, ppm) displayed signals at 4.07 (s, 2H, CH₂), 7.37–8.34 (m, 13H, aromatic H), 8.40 (s, 1H, CH=N), 8.62 (s, 1H, pyrazole C5-H), 8.89 (s, 1H, aromatic H), and 11.60 (broad s, 1H, NH). ¹³C NMR (100 MHz, DMSO-d₆, δ, ppm) exhibited resonances at 56.5, 114.6, 117.7, 119.1, 120.4, 124.1, 125.3, 127.4, 128.4, 128.9, 129.4, 130.7, 131.2, 132.6, 136.3, 137.0, 139.3, 142.4, 144.0, 147.0, 148.4, 148.6, 151.8, 160.0, 161.2. Elemental analysis for C₂₅H₁₉N₇O₂S (M = 481.53 g/mol) gave calculated values of C, 62.36%; H, 3.98%; N, 20.36%, whereas the experimentally found values were C, 62.27%; H, 3.94%; N, 20.34%.

## **Structural confirmation of inhibitors**


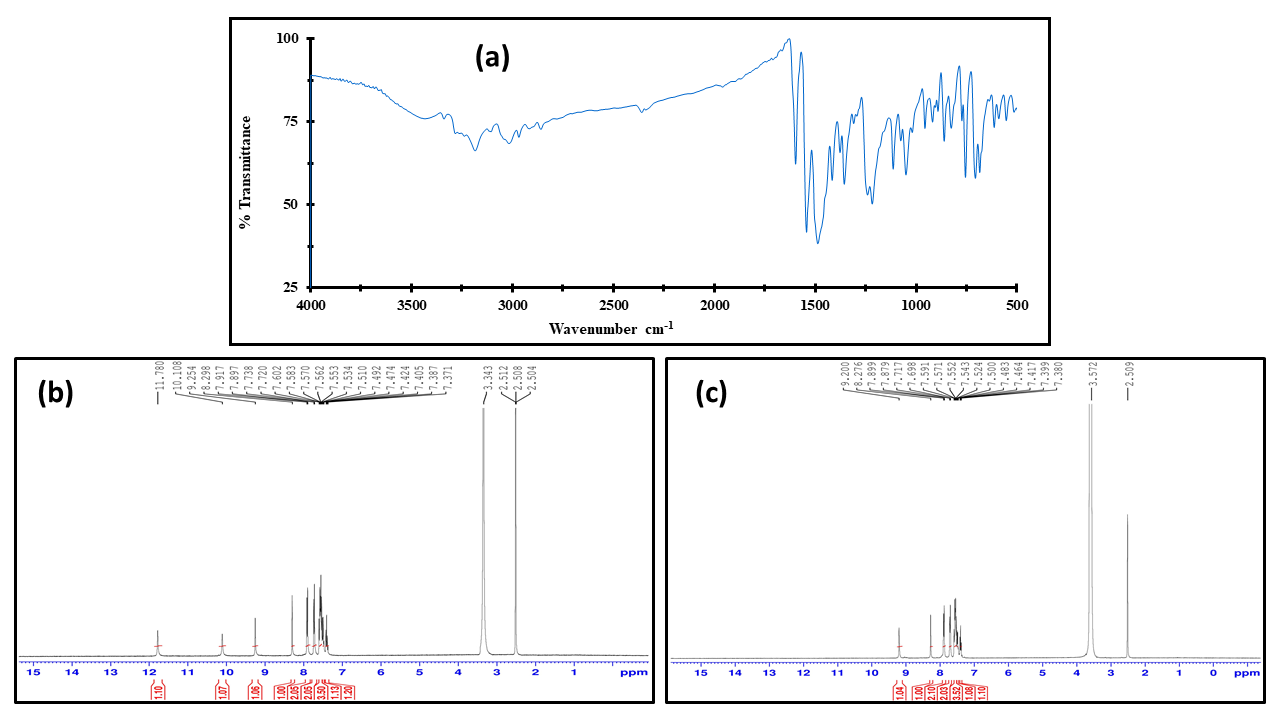


**Fig. S1**. Spectral data of P6 inhibitor, where (a) IR spectrum, (b) ^1^H NMR spectrum (DMSO-*d*_6_), and (c) ^1^H NMR spectrum (DMSO-*d*_6_+D_2_O).


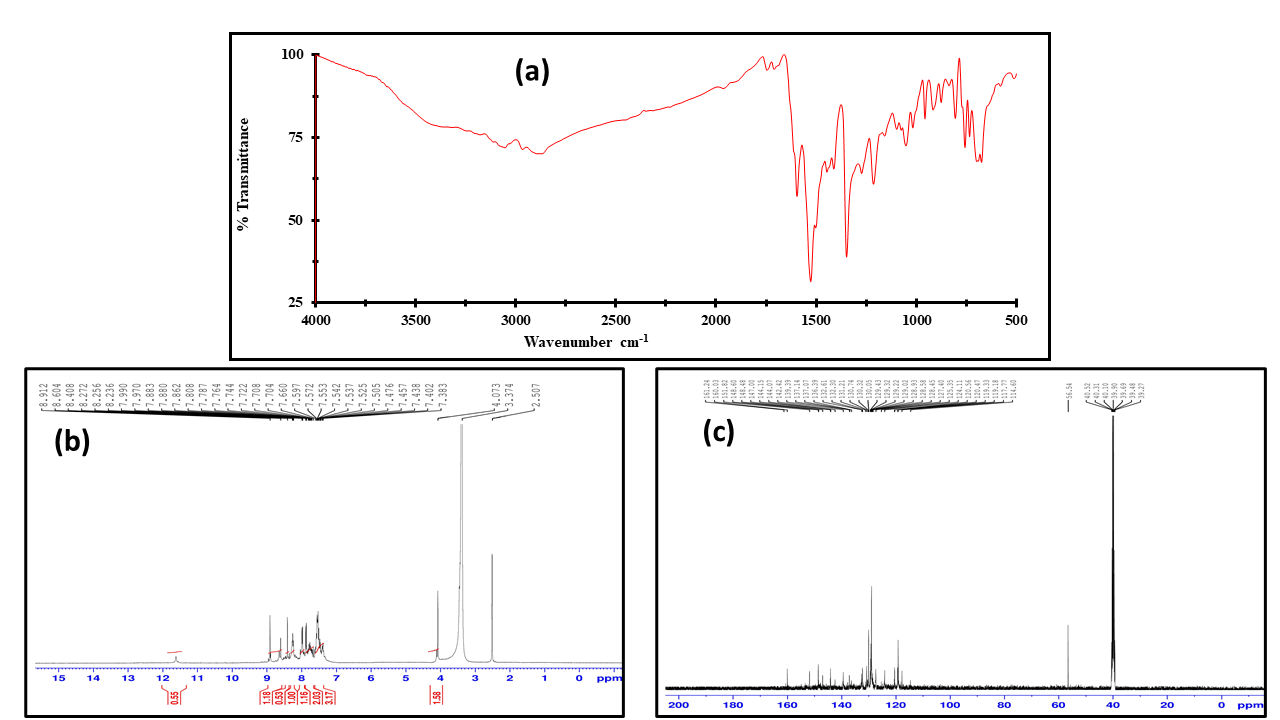


**Fig. S2**. Spectral data of P8 inhibitor, where (a) IR spectrum, (b) ^1^H NMR spectrum (DMSO-*d*_6_), and (c) ^13^C NMR spectrum (DMSO-*d*_6_).

## **SI (2). Effect of immersion time on corrosion behavior**

The stability of the protective films formed by **P6** and **P8** on carbon steel was evaluated using EIS over an immersion period of up to 24 *h* (Table S1, Fig. S3). In the absence of inhibitors, the polarization resistance decreases steadily from 22.26 Ω·cm² at 0.5 h to 7.91 Ω·cm² after 24 *h*, indicating continuous surface degradation in the aggressive acidic medium. This trend is consistent with the reduction in Nyquist semicircle diameter, reflecting enhanced charge transfer and accelerated corrosion. In contrast, both inhibitors maintain significantly higher *R_p_* values throughout the immersion period. For P6, *R_p_* reaches 497.4 Ω·cm² after 2 *h* and remains as high as 288.3 Ω·cm² after 24 *h*, with inhibition efficiencies exceeding 97%. Similarly, P8 shows sustained protection, with inhibition efficiencies above 92% after 24 *h* (Table S1). The Nyquist plots retain their depressed semicircular shape at all immersion times, indicating that the corrosion process remains charge-transfer controlled. Overall, both inhibitors exhibit good long-term stability, with P6 showing slightly superior performance.

**Table S1**. Variation of electrochemical impedance parameters of CS in 1.0 M HCl solution with immersion time in the absence and presence of P6 and P8 inhibitors.

| *Inh*. | *Time (h)* | *R_s_, (Ω.cm^2^)* | *R_P_ (Ω.cm^2^)* | *Ɵ* | *𝜂 %* |
| --- | --- | --- | --- | --- | --- |
| Blank | 0.5 | 1.463 | 22.26 | — | — |
|  | 1 | 3.792 | 18.2 | — | — |
|  | 2 | 1.599 | 16.87 | — | — |
|  | 4 | 3.472 | 15.65 | — | — |
|  | 6 | 3.7 | 12.3 | — | — |
|  | 24 | 3.408 | 7.908 | — | — |
| P6 | 0.5 | 5.808 | 559.8 | 0.96 | 96 |
|  | 1 | 10.32 | 507.2 | 0.9641 | 96.41 |
|  | 2 | 11.08 | 497.4 | 0.9661 | 96.61 |
|  | 4 | 7.94 | 482 | 0.9675 | 96.75 |
|  | 6 | 6.533 | 475.7 | 0.9741 | 97.41 |
|  | 24 | 7.338 | 288.3 | 0.9723 | 97.23 |
| P8 | 0.5 | 5.83 | 335 | 0.9336 | 93.36 |
|  | 1 | 7.585 | 249.8 | 0.9271 | 92.71 |
|  | 2 | 6.577 | 194.7 | 0.9134 | 91.71 |
|  | 4 | 6.901 | 169 | 0.9074 | 90.74 |
|  | 6 | 6.867 | 129.3 | 0.9049 | 90.49 |
|  | 24 | 7.618 | 100.1 | 0.921 | 92.1 |

**
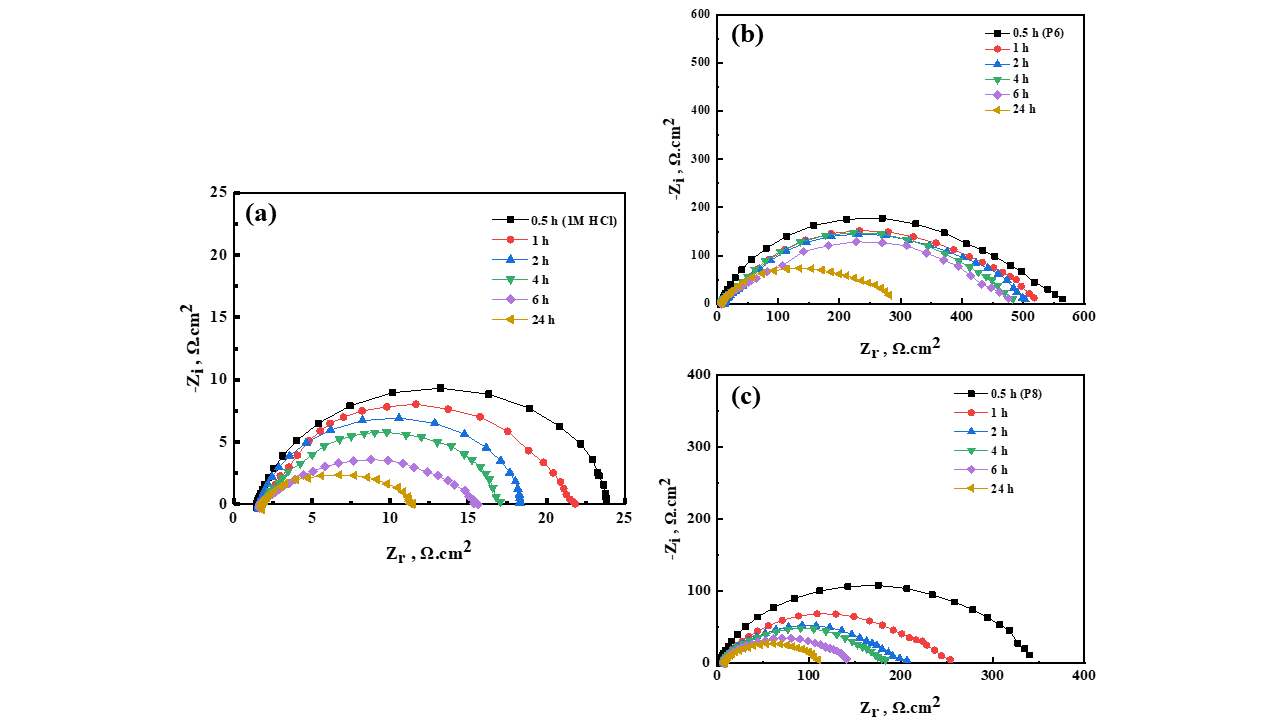
**

**Fig. S3. Nyquist impedance plots of CS in 1.0 M HCl solution recorded at different immersion times: blank solution (a), in the presence of P6 (b), and in the presence of P8 (c).**


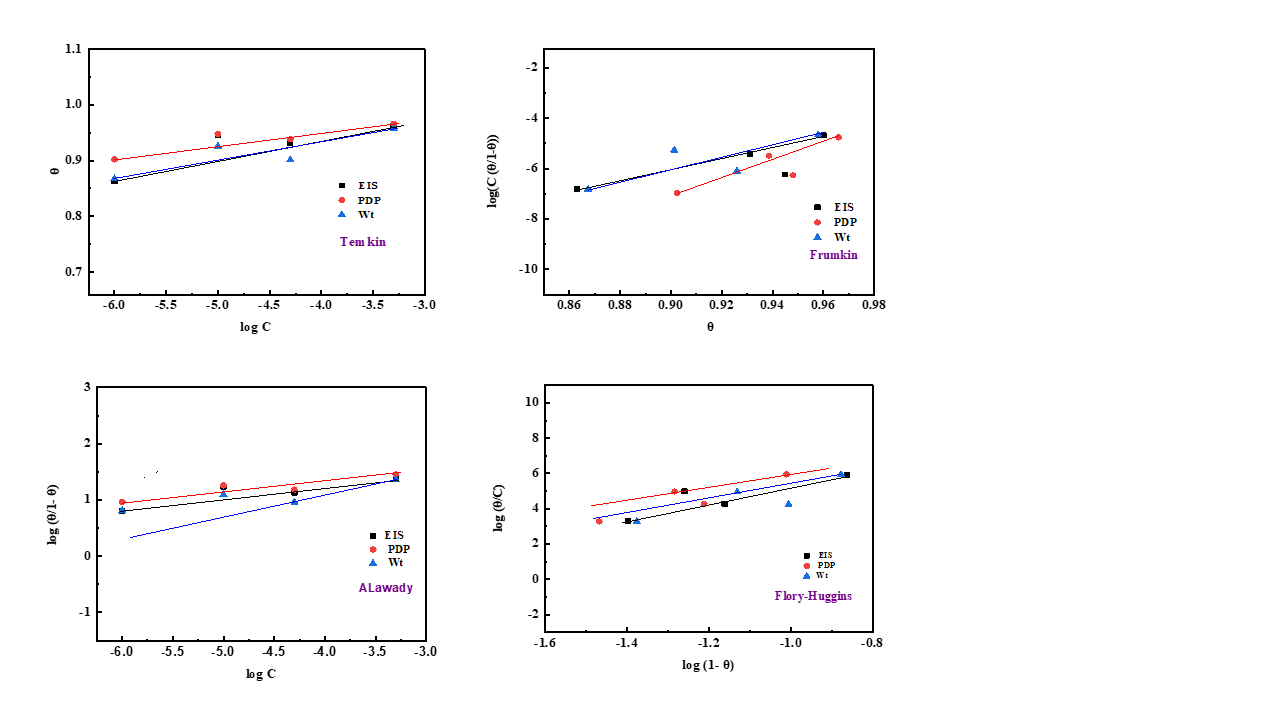


**Fig. S4. Adsorption isotherm plots describing the adsorption behavior of P6 inhibitor on the CS surface in 1.0 M HCl solution.**


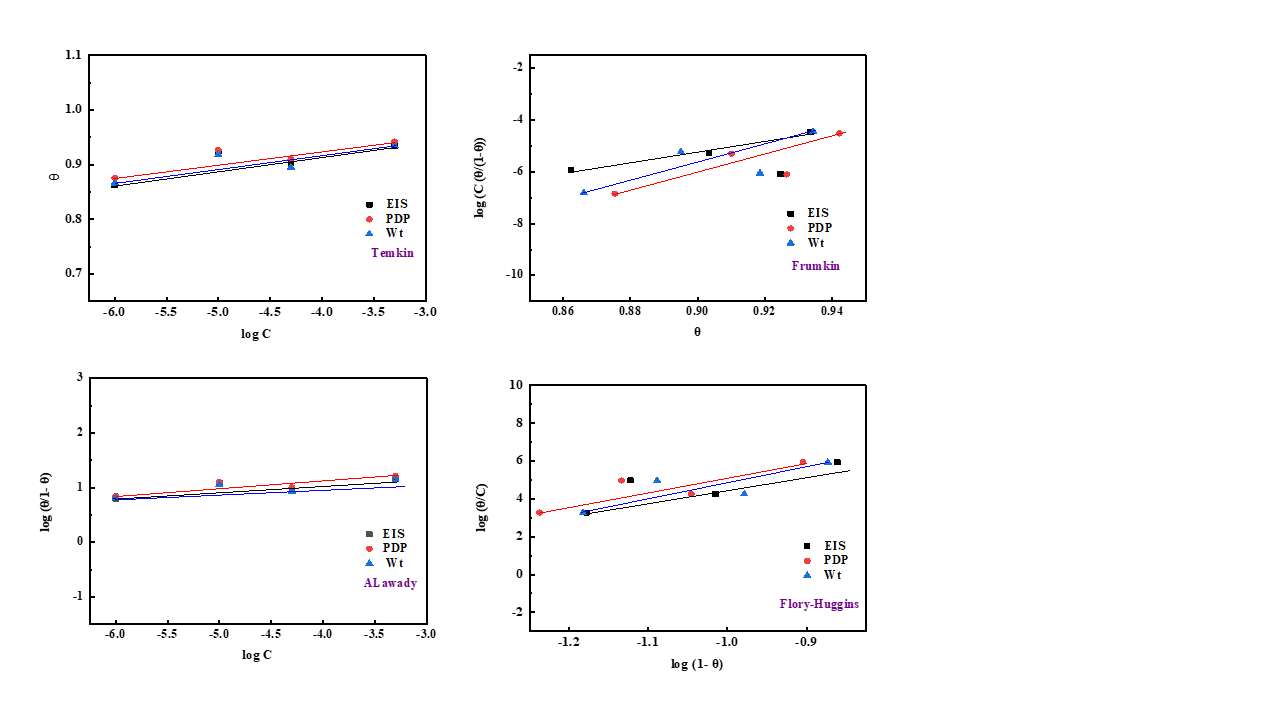


**Fig. S5. Adsorption isotherm plots describing the adsorption behavior of P8 inhibitor on the CS surface in 1.0 M HCl solution.**

**
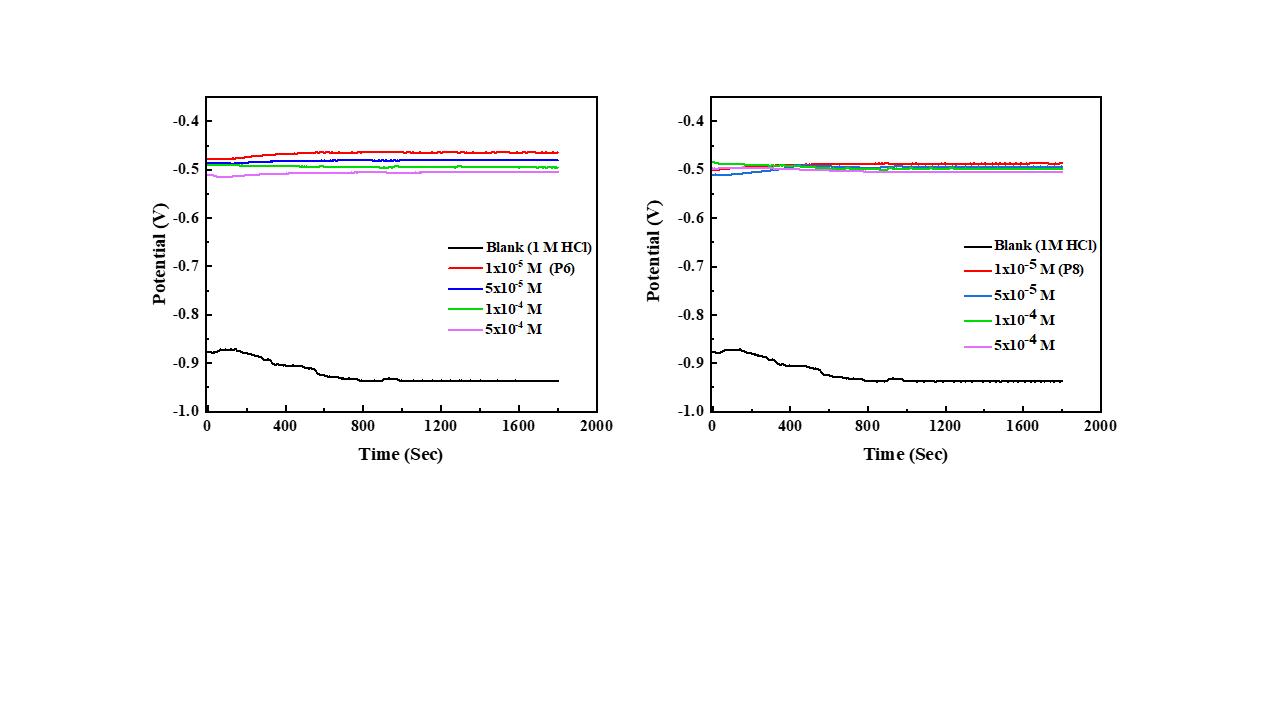
**

**Fig. S7.** OCP vs. time for CS in 1.0 M HCl in the absence and presence of different concentrations of P6 and P8 at room temperature.

**Table S2**. Comparison between the inhibition efficiency of the prepared inhibitors (**P6** & **P8**) and other investigated inhibitors for CS in 1.0 M HCl solution.

| *Inh.* | *Structural Class* | *Medium* | *Optimum concentration* | *𝜂 %* | *Reference* |
| --- | --- | --- | --- | --- | --- |
| BBPA | Bipyrazole derivative | 1.0 M HCl | 5x10^-4^ M | 87.0 | 39 |
| BM-01 |  |  | 1x10^-3^ M | 90.38 | 40 |
| Pyrazole derivative (I/II) | Pyrazole-thiophene derivative |  | 5x10^-4^ M | 93.0–93.5 | 41 |
| Bis-Pyr | Bis-pyrazoline derivative |  | 400 ppm | 90.41 | 42 |
| DPCM | Pyrazole derivative |  | 400 ppm | 89.5 | 15 |
| 6a | Pyrazolone-sulfonamide derivative |  | 500 ppm | 94.81 | 26 |
| 2PzH | Bipyrazole derivative |  | 1x10^-3^ M | 95.7 | 43 |
| 2PzMe |  |  |  | 95.9 |  |
| SHA | benzimidazole derivatives |  | 1x10^-3^ M | 93.7 | 44 |
| P6 | Bis-pyrazole derivative |  | 5x10^-4^ M | 96.00 | This work |
| P8 | Pyrazolyl-thiadiazine derivative |  |  | 93.36 |  |
